# Forensic Mass Spectrometry: Scientific and Legal Precedents

**DOI:** 10.1021/jasms.3c00124

**Published:** 2023-06-05

**Authors:** Glen P. Jackson, Mark A. Barkett

**Affiliations:** † Department of Forensic and Investigative Science, West Virginia University, Morgantown, West Virginia 26506-6121, United States; ‡ C. Eugene Bennett Department of Chemistry, West Virginia University, Morgantown, West Virginia 26506, United States; § Dover Chemical Company, Dover, Ohio 44622, United States

## Abstract

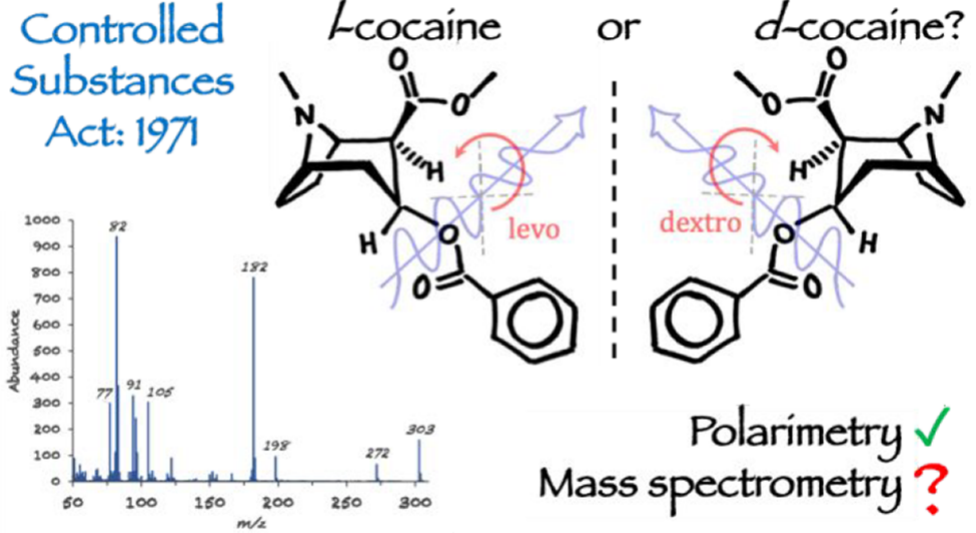

Mass spectrometry has made profound contributions to the criminal justice system by providing an instrumental method of analysis that delivers exquisite analytical figures of merit for a wide variety of samples and analytes. Applications include the characterization of trace metal impurities in hair and glass to the identification of drugs, explosives, polymers, and ignitable liquids. This review describes major historical developments and, where possible, relates the developed capabilities to casework and legal precedents. This review also provides insight into how historical applications have evolved into, and out of, modern consensus standards. Unlike many pattern-based techniques and physical-matching methods, mass spectrometry has strong scientific foundations and a long history of successful applications that have made it one of the most reliable and respected sources of scientific evidence in criminal and civil cases. That said, in several appellate decisions in which mass spectrometric evidence was challenged but admitted, decisions sometimes still went against the mass spectrometric data anyway, which goes to show that mass spectrometric evidence is always just one piece of the larger legal puzzle.

## Introduction

When conducting historical research on legal precedents, it is almost impossible to unearth cases in which mass spectrometric evidence was simply admitted and used to resolve a dispute. The reason is that, unless a journalist in the courtroom reports on the specific details of the case,^1,2^ evidential details, like analytical results, typically are not searchable in the public domain. In contrast, appellate decisions at all levels, especially state and federal, tend to be published and freely available in online databases such as Nexis Uni (formerly Lexis Nexis). The legal precedents identified here are therefore identified in three ways: (1) by reference in the traditional peer-reviewed literature, (2) from newspaper reports, and (3) from various searches of appellate decisions in Nexis Uni.

**Figure 1:**
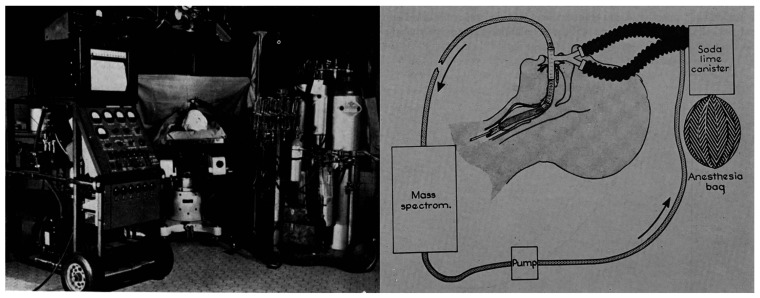
In 1950, Alfred O. Nier developed this portable mass spectrometer for real-time monitoring of exhaled gases from the trachea of patients under anesthesia. The peripherally related legal matter ruled in favor of the U.S. Tax Court that a medical fellow on the project should have reported his fellowship award as taxable income. Reproduced with permission from ref (6). Copyright 1950 American Society of Thoracic Surgery.

One of the earliest references to mass spectrometry in the legal literature is in 1950, when H. W. Washburn appealed the rejection of claims on his patent application for an ion extraction potential in an EI source and mixture analysis using mass spectrometry.^3^ Since then, most of the documented legal history of mass spectrometry has involved patent disputes and is beyond the scope of this review. Another theme among the early legal cases is customs infringements and other issues related to the manufacture and distribution of instruments. This review will not cover these cases either. One exception to including a case report without an analytical result is an interesting court decision on a personal tax matter. In 1948, A. O. Nier had reported on a home-built portable mass spectrometer for monitoring process gases in real time,^4^ and within a couple of years he, and others, had made a portable version of the instrument for monitoring exhaled gases of patients undergoing anesthesia (Figure 1).^5−7^ The appellate ruling, in favor of the US Tax Commissioner, held that a collaborating medical fellow on the project should have reported his fellowship award of $2,200 in 1953 as taxable income.^8^

The remaining legal examples of mass spectrometry in this review will focus on those involving analytical results. This review aims to provide historical perspective rather than comprehensive coverage. Reviews describing comprehensive coverage of research developments can be found in reviews by J. Yinon and G. P. Jackson et al., among others.^9−11^

One of the first mass spectrometers in an actual forensic laboratory was in Birmingham, England in 1973. J. A. Zoro and K. Hadley reported the details of the workload of this mass spectrometer in a fascinating summary in 1976 (Table 1).^12^ The greatest proportion of cases involved the analysis of drugs, both in bulk form and in human bodily fluids. More than 50 years later, the Office of Justice Statistics showed that drug identifications remain the most frequently submitted evidence request in a typical forensic laboratory.^13^

**Table 1:** Distribution of Case Types of the First Year of Operation (1973) of a Mass Spectrometer in the Home Office Central Research Establishment in Birmingham, UK^a^

Number of cases	Type of case
59	Illegal possession of drugs
47	Suspicious death
18	Explosives
17	Arson
10	Miscellaneous
8	Administration of noxious substance
7	Driving under the influence of drugs
7	Malicious damage
4	Documents
2	Biology

a Reproduced with permission from ref (12). Copyright 1976 Forensic Science Society.

## Drugs and Toxicology

### The Beginnings

As indicated in Table 1, forensic applications of mass spectrometry most frequently involve the analysis of drugs, drug metabolites and drug paraphernalia. Organic mass spectrometry began in 1929 when W. Bleakney developed the electron ionization (née impact) source for the analysis of gases and inorganic vapors.^14^ He extended the work to simple volatile hydrocarbons in the late 1930s.^15^ The first commercial vendor was the Consolidated Electric Company (CEC) in the mid-1940s. The instruments were large, expensive, difficult to operate,^16^ and there was almost no guidance for spectral interpretation until the mid-1950s when J. H. Beynon and F. W. McLafferty published helpful expositions that described mechanisms, trends, and tips for interpreting mass spectra of organics.^17−20^ In the 1960s, the groups of K. Biemann and C. Djerassi were prolific in applying mass spectrometry to the analysis of natural products and botanical extracts, including cannabis and tropane alkaloids related to cocaine.^21−26^ Other groups also contributed to the growing collection of tropane alkaloid data, including from drug seizures.^27−29^

In 1968, R. J. Martin and T. G. Alexander at the U.S. Food and Drug Administration (FDA) explained how they used cracking patterns and high resolution mass spectrometry (HRMS) to identify the hallucinogen dimethyltryptamine (DMT) in a casework sample.^30^ They reported that “a problem that would have constituted a major research project a few years ago was reduced to an exercise problem in spectroscopic identification.”^30^ The same year, R. T. Coutts and R. A. Locock characterized eight common barbiturates and their mixtures in pills and capsules.^31^ Between 1968 and 1970, S. W. Bellman and co-workers at the FDA used an Associated Electrical Industries MS-12 mass spectrometer to identify several hallucinogenic drugs via a direct insertion probe.^30,32,33^ These initial applications included mescaline, psilocin, psilocybin, and analogs of lysergic acid diethylamide (LSD), among others. Other groups quickly followed suit.^34,35^

**Figure 2:**
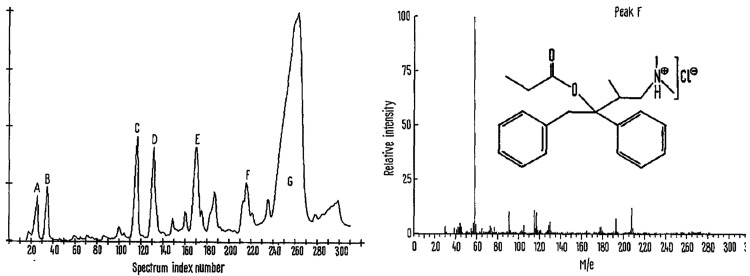
Total ion chromatogram of a urine extract showing Darvon (peak F) and various metabolites. Reproduced with permission from ref (36)**.**(36) Copyright 1970 Springer Nature.

At the Massachusetts Institute of Technology (MIT) in 1970, J. Althaus et al. used a computer-assisted gas chromatography–mass spectrometry (GC-MS) to detect Darvon and its metabolites in the urine of a victim of a suspected overdose patient (Figure 2).^36^ Data included low-resolution GC-MS data and high resolution mass spectrometric data.

Unlike today’s backlogs, the case was solved in about one day, albeit by a team of graduate-level MIT scientists. By 1971, H. M. Fales’ group at the National Institutes of Health (NIH) had solved more than 100 cases involving drug overdoses using GC-MS and computer-assistant database searching.^37^ Samples included both blood serum and stomach contents. The same year, M. Blomquist et al. provided fascinating details of 13 randomly selected cases in which GC-MS had helped to identify drugs in various biological tissues at a government laboratory in Sweden.^38^ In 1972, R. F. Skinner et al. reviewed the status of GC-MS for forensic toxicology.^39^ His group had used a new Finnigan 1015C GC-MS, and most of the reported casework involved the detection of barbiturates in various body fluids “within 15 min,” assuming the instrument was in standby mode.^39^

Around the same time, S. Agurell and colleagues, in Sweden, had also used GC-MS and mass fragmentography to identify drugs in various cases.^40,41^ Applications included precursors of mescaline in Peyote cactus and various drugs in the blood of subjects who had recently smoked them. The *New York Times* reported on a presentation from their group in which a GC-MS assay for Δ^9^-THC in human blood was sensitive enough to detect if someone had smoked “one half-billionth of a gram.”^1^ (The original report used the name delta-1-THC, which is based on the monoterpene numbering system. Modern convention uses the benzopyran numbering system; delta-9-THC.)

### In Vivo

In 1972, D. E. Green showed the potential of mass fragmentography to detect alcohol in circulating blood in vivo and in real-time. In addition, he used modified sampling devices to identify drugs on surfaces with minimal sample preparation besides rendering them neutral to increase their vapor pressures.^42^ Although the ability to detect chemicals from human skin in real time sounds cutting edge, even by today’s standards, B. Adamczyk et al. had already shown the ability to detect gaseous excretions from human skin using a continuously monitoring mass spectrometer in 1966.^43^ In the early 2000s, ambient sampling mass spectrometry witnessed an enormous resurgence following the introduction of both desorption electrospray ionization (DESI) and direct analysis in real time (DART).^44−46^ Despite the amazing examples in the 1950s, 1960s, and 1970s, real-time clinical applications of mass spectrometry have taken a long time to mature.^6,7,42,43^

### In the Crime Lab

In 1973, B. Stein et al. published a review of the procedures and analyst qualifications in more than 100 forensic laboratories in the United States.^47^ The report provides shocking examples of expert testimony in hundreds of cases by unqualified analysts.^47^ For example, more than 60% of those using spectroscopic methods to identify drugs had never taken a college course on the topic. Only two forensic laboratories out of 123 that were surveyed had used mass spectrometry for casework, and when Stein et al. asked the respondents which new instruments they would like if the money were available, GC-MS was the most desired piece of new equipment. However, only nine out of 123 laboratories said they would like one. It is hard to imagine modern lab directors being so unenthusiastic about the possibility of a new and free mass spectrometer. Still, the advent of commercial GC-MS instruments in the early 1970s meant that mass spectrometry was quickly gaining popularity.

In 1973, R. Saferstein and J.-M. Chao reported on the use of chemical ionization (CI), which had been introduced by M. S. B. Munson and F. H. Field in 1966, to analyze drugs and drug mixtures.^48,49^ By 1974, I. Jardine and C. Fenselau added charge exchange ionization to the analysis of drugs, and many other groups were adding to the forensic mass spectrometry literature.^9,50^ Many early forensic applications were captured in Fenselau’s comprehensive review of GC-MS in 1974.^51^ Toxicologists soon considered GC-MS a mainstay for identifying trace levels of drugs and metabolites in biological samples.^52,53^

### Mass Spectrometry Defended in Court

In 1977, mass spectrometric data from the U.S. Environmental Protection Agency (EPA) was admitted as evidence in *Citizens Against Toxic Sprays v. Bergland.* The case involved the detection of the pesticide tetrachlorodibenzo-*p*-dioxin (TCDD) in animal tissues after grazing in the Siuslaw National Forest.^54^ In 1978, The New York Times reported that a judge had ruled to allow mass spectrometric test results as evidence in a capital murder case in which mass spectrometry had identified curare in the blood of three victims where radioimmunoassay and chromatography had failed.^2^ After the second longest murder trial in US history, the defendant, Dr. “X”, was acquitted of murdering five victims anyway.^55^

### Bringing Home the Bacon

In 1978, GC-MS results were also admitted in *American Meat Institute v. Bergland* to determine whether or not bacon had been adulterated such that it contained elevated levels of nitrosamines after cooking.^56^ In possibly the best smelling laboratory procedure ever, analysts on the case had to first cook the bacon to prepare it for analysis. GC-MS was used specifically because it was “widely regarded as the best available technology.”^56^

**Figure 3:**
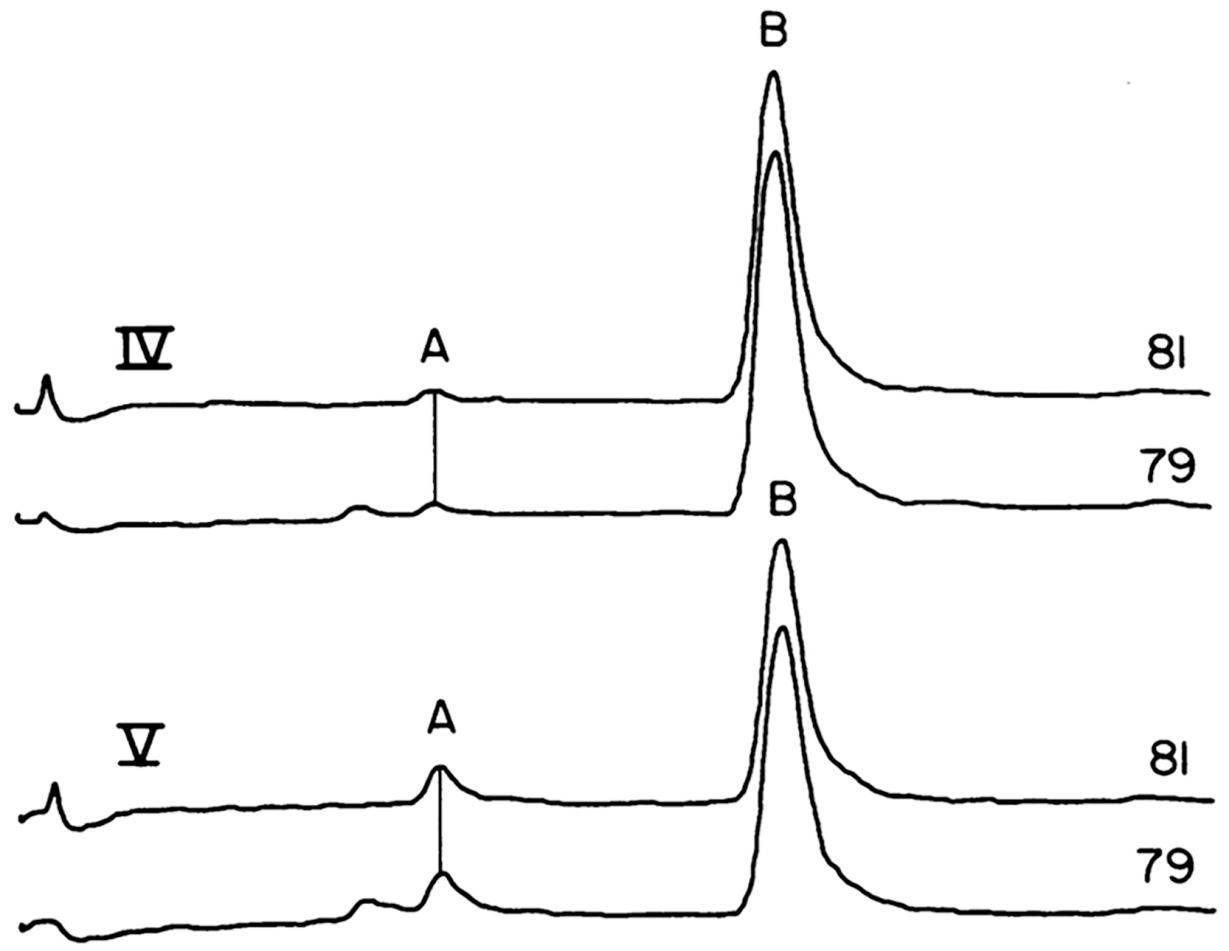
Extracted ion profiles for APCI-MS chromatograms of bromine isotopes at *m*/*z* 79 and 81 to show the exposure of children to the flame-retardant Tris-BP through urinalysis of the metabolite 2,3-dobromopropanol (Peak A) after their pajamas had been laundered for ∼5 months. Peak B is an internal standard. Figure adapted with permission from ref (57). Copyright 1978 The American Association for the Advancement of Science.

In the 1970s, negative CI-MS helped overturn a ruling that ultimately led to the conviction of a company that was manufacturing a flame-retardant for children’s pajamas. Atmospheric pressure negative-CI-MS detected 2,3-dibromopropanol, which is a metabolite of the flame retardant tris(2,3-dibromopropyl) phosphate (Tris-BP), in the urine of children who had worn the flame-resistant pajamas (Figure 3).^57,58^

In a review article on urinalysis in 1979, a probation officer named P. J. Bigger stated that although mass spectrometry was “the most sensitive and specific technique available,” it was “too expensive and too slow to be commonplace.”^59^ Thankfully, budgets expanded, and the arrival of autosamplers in the 1990s meant that GC-MS instruments could operate all night while analysts slept.

### Cocaine Isomers Cause Headaches

By the end of the 1970s more than a dozen groups had contributed to the analysis of cocaine and its metabolites.^29,37,49,50,60−64^ In 1978, R. W. Kondrat and R. G. Cooks were arguably the first to apply tandem mass analysis to a forensic application when they fragmented cocaine from a complex mixture of coca leaf extract without the need for wet-chemical isolation or chromatographic separation.^65^

After passage of the Controlled Substances Act (CSA) of 1970,^66^ arguments continued over the need to distinguish harmless d-cocaine from the active drug, l-cocaine.^67−70^ For the next decade, if GC-MS was applied to the analysis of cocaine at all, it had to be accompanied by a polarimetry test to address the isomeric form.^71,72^ For most of the 1970s it was common for analysts to suffer embarrassing testimonies about cocaine isomers (there are only two common isomers, but eight total)^72^ until 1981 when an FBI analyst saved the day by noting that because plants make exclusively l-cocaine and chemical synthesis results in a racemic mixture of d- and l-cocaine, d-cocaine “had also never been seen apart from l-cocaine.”^73^ The judge accepted his insight, and, thanks to his testimony, analysts no longer had to identify the isomeric form of cocaine. From ∼1981 onward, analysts dropped polarimetry tests and relied instead on GC-MS to identify cocaine.

At the 1972 Olympics in Munich, GC-MS screening found seven adverse findings from 2079 tests of athletes’ blood and urine.^74,75^ In 1994, M. Becchi et al. showed that GC-combustion-isotope ratio mass spectrometry (GC-C-IRMS) could distinguish exogenous and endogenous testosterone and thereby prove that elevated levels of testosterone were caused by doping.^76^ IRMS is still used by the world antidoping agency (WADA), among others, to help discriminate natural versus exogenous sources of hormones.^77^

### Hashing out Problems with Marijuana

In 1974, D. S. Fullerton and M. G. Kurzman were concerned that too many suspects were being wrongfully convicted for possession of marijuana based on unselective color tests, so they wrote a comprehensive report that called for the addition of confirmatory methods like GC-MS for the identification of marijuana.^78,79^ Others agreed. In its 1978 decision in *Minnesota v. Vail*, the Supreme Court of Minnesota rejected the State’s chemical evidence of marijuana because it was not selective enough.^80^ The Court noted that microscopic analysis, when combined with GC-MS, could identify marijuana beyond a reasonable doubt, but GC-MS was not used, so the test was not sufficiently reliable. In their report, Fullerton and Kurzman also called for a better definition of illegal marijuana, which did not come for more than four decades until the passage of the Agriculture Improvement Act of 2018, also known as the Farm Bill.^79,81^ The Farm Bill updated language in the CSA to define illegal forms of cannabis and THC-containing products as those containing more than 0.3% by weight of delta-9-THC.^66^ Curiously, the latest version of ASTM E2329-17: Standard Practice for Identification of Seized Drugs, still allows analysts to not use a confirmatory test like GC-MS in the identification of marijuana, but the standard has not been updated since the passage of the Farm Bill.

**Figure 4:**
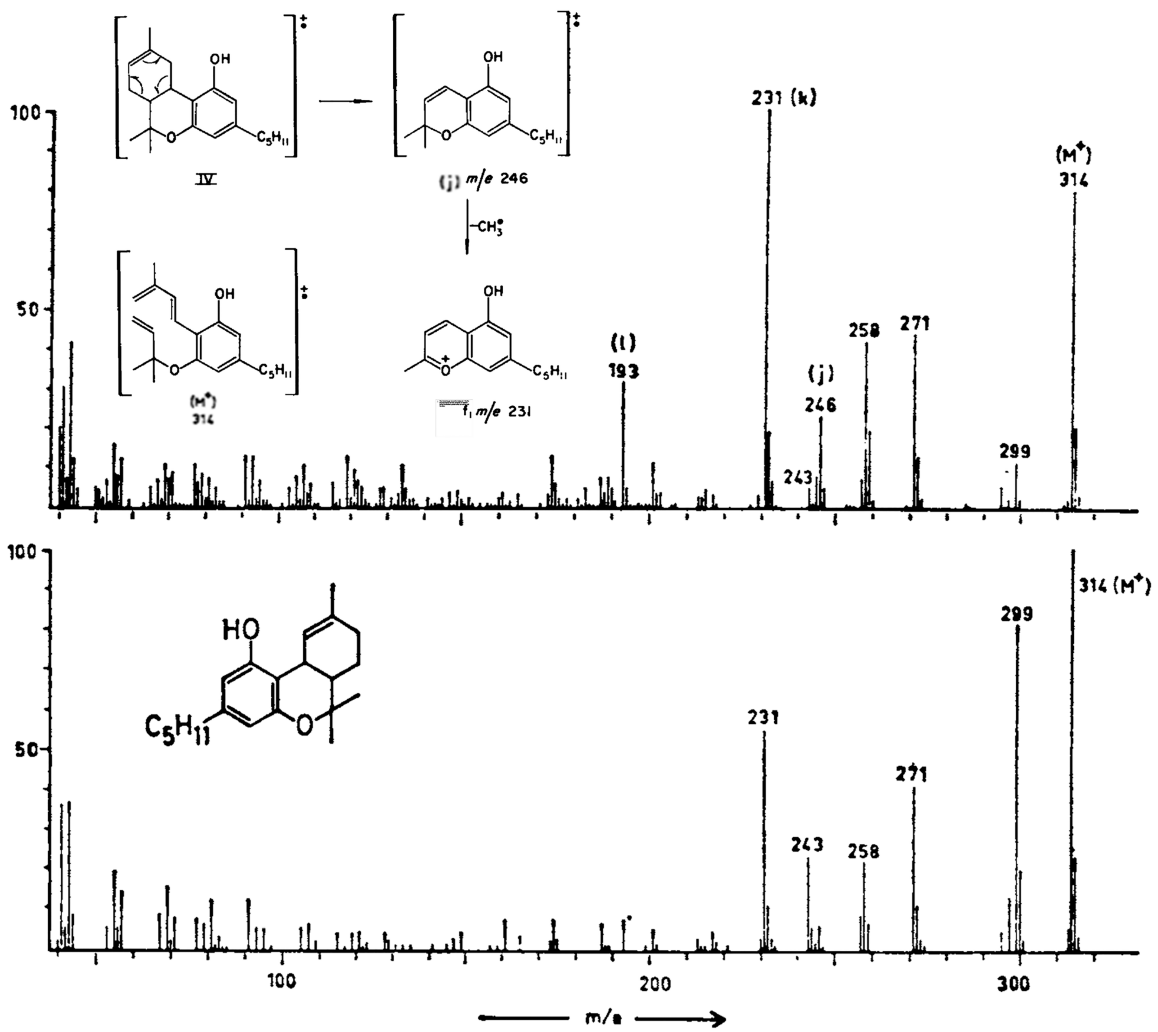
Mass spectra of delta-8-THC (top) and delta-9-THC (bottom) to show that only the delta-8 isomer undergoes the retro-Diels–Alder rearrangement (j) to form the fragment at *m*/*z* 246. Adapted with permission from refs (22) and (87). Copyright 1965 Elsevier and 1987 Wiley Periodicals, Inc., respectively.

The specificity of the wording regarding the single cannabinoid delta-9-THC in the Farm Bill led certain entrepreneurs to erroneously believe that positional isomers like delta-8-THC were legal.^82^ Delta-8-THC is readily formed via acidic treatment of Delta-9-cannabidiol (CBD), which is one of the most abundant cannabinoids in unregulated hemp oil.^83^ However, isomerization of the double bond during acid-cyclization means that isomers like delta-9-THC and delta-10-THC are usually formed as byproducts in the conversion process and therefore commonly found in final products containing delta-8-THC.^84^ Interestingly, when the structures of the various cannabinoids were first confirmed in the mid 1960s through isolation and partial synthesis, Y. Gaoni and R. Mechoulam obtained their semisynthetic delta-9-THC reference through acidic treatment of delta-9-CBD; the same treatment used today to produce delta-8-THC.^85,86^ In the first two descriptions of delta-9-THC, mass spectrometry was not used to support the NMR and IR characterization.^85,86^ If mass spectrometry had been used, the EI-mass spectra of delta-8-THC and delta-9-THC would have readily resolved the double bond position because, as T. B. Vree first showed in 1977, only the delta-8 isomer can undergo a retro-Diels–Alder rearrangement to form a fragment at *m*/*z* 146 (Figure 4).^84,87,88^ Retro-Diels–Alder rearrangement of delta-9-THC does occur, but it does not lead to separation of the products, so the peaks at *m*/*z* 314 and 299 (−15 Da) are enhanced relative to the same peaks for delta-8-THC.

### The China White Puzzle

In 1981, T. C. Kram et al. at the DEA reported on the use of GC-EI-MS and GC–CI-MS in combination with NMR and FITR to help elucidate the structure of the extremely active substance in a seizure of China White in California.^89^ EI-MS provided a spectrum with the heaviest fragment at *m*/*z* 259, which was presumed to be the molecular ion. However, CI-MS provided the missing clue by providing a protonated molecular ion at *m*/*z* 351, so the peak at *m*/*z* 259 was easily explained by the loss of a benzyl group (91 Da) from the unobserved EI-molecular ion at *m*/*z* 350. Additional reasoning and NMR evidence helped complete the structural elucidation to be alpha-methylfentanyl: the first of what are now hundreds of known fentanyl analogs that continue to plague the US with accidental overdoses.

### A Bone to Pick with Eminent Witnesses

Mass spectrometry does not always fare well in court. In *US v. 2,116 Boxes of Boned Beef* (co-defendants included 541 boxes of offal), the U.S. District Court of Kansas was not impressed by the GC-MS evidence in the case.^87,90^ The case concerned the alleged adulteration of beef with the hormone diethylstilbestrol. Regarding the eminent witnesses, the Court lamented that “they are disregarded as being of any scientific assistance to the Court. Simply stated, a review of these exhibits suggests that the experts can read into them about what they want to read, the Court perceiving nothing and is totally helpless.”^90^ After another criticism of the experts’ tortuous descriptions, the Court also noted that “hopefully, such an observation does not offend the scientific world, but it is submitted here to express in part the Court’s quandary in this most technical field.”^90^ This self-admission by the Court of its incongruency with the experts is a reminder to all expert witnesses of the need to explain their science well if it is to have any value at all.

### Substantial Problems with the Analogs Act

In 1986, the Controlled Substance Analogue Enforcement Act (CSAEA),^91^ also known as the Analogs Act, was signed into law to control compounds that were “substantially similar” to drugs that were scheduled in the original CSA.^66^ In its 1992 decision in *United States v. Forbes*, the District Court of Colorado ruled that CSAEA’s language was “unconstitutionally vague” and that alpha-ethyltryptamine (AET) was not substantially similar to the scheduled drugs dimethyltryptamine (DMT) or diethyltryptamine (DET).^92^ Instead of clarifying the wording of the law, the DEA simply added AET to the list of scheduled drugs. Ten years later, a circuit-court judge in *United States v. Washam* found the same questionable language in CSAEA to be valid.^93^ He ruled in favor of the district court and the prosecution that 1,4-butanediol was an analog of gamma hydroxybutyrate (GHB), and the defendant’s conviction was upheld. The same year, in *United States v. Klecker*,^94^ a judge also found the language to be lawful and agreed that government had shown substantial similarity between the structures and effect on humans of the seized substance Foxy and the scheduled drug diethyltryptamine (DET). Many additional cases have now upheld the language and constitutionality of the Analogs Act.

In 1991, a mother was found guilty of poisoning her 5-month-old child with ethylene glycol based on GC with a flame ionization detector (FID).^95^ She gave birth to a second child in prison, and after that child also became ill, doctors were able to diagnose a genetic disorder called methylmalonic acidemia. However, reanalysis of serum from the first child using GC-FID again revealed ethylene glycol, and because methylmalonic acidemia does not cause a buildup in ethylene glycol, the mother was still charged with poisoning her first child. A toxicologist named Jim Shoemaker developed a more selective GC-MS approach that proved that the toxin was in fact propionic acid, which could be linked to the genetic disorder.^96^ Thankfully, the mother was ultimately exonerated.^95^

## Arson

In 1959, a firearms technician at the Chicago police crime lab named Joseph Nicol suggested that crime laboratories should collaborate with universities or oil companies to use their GC-MS instruments for important arson cases.^97^ As mentioned earlier, J. A. Zoro and K. Hadley’s review in 1976 recommended the use of GC-MS over the less-selective GC-FID, which was the existing state-of-the art for identifying the presence of ignitable liquids (née accelerants).^12,98,99^ In *Montana v. Burtchett*, the supreme court found merits in the prosecutions use of GC-FID for the identification of gasoline residues in a structure fire.^98^ In reaching their decision, they noted that that the chemist who examined the samples testified that he readily detected marked, distinguishable difference between accelerants and other nonvolatile petroleum distillates that the appellant contended were in the floor. The Court made the point of noting that “his testimony was lengthy and technical but that is the thrust of it,”^98^ which serves as a reminder to keep expert-witness testimony succinct.

### Fuel on the Fire?

**Figure 5:**
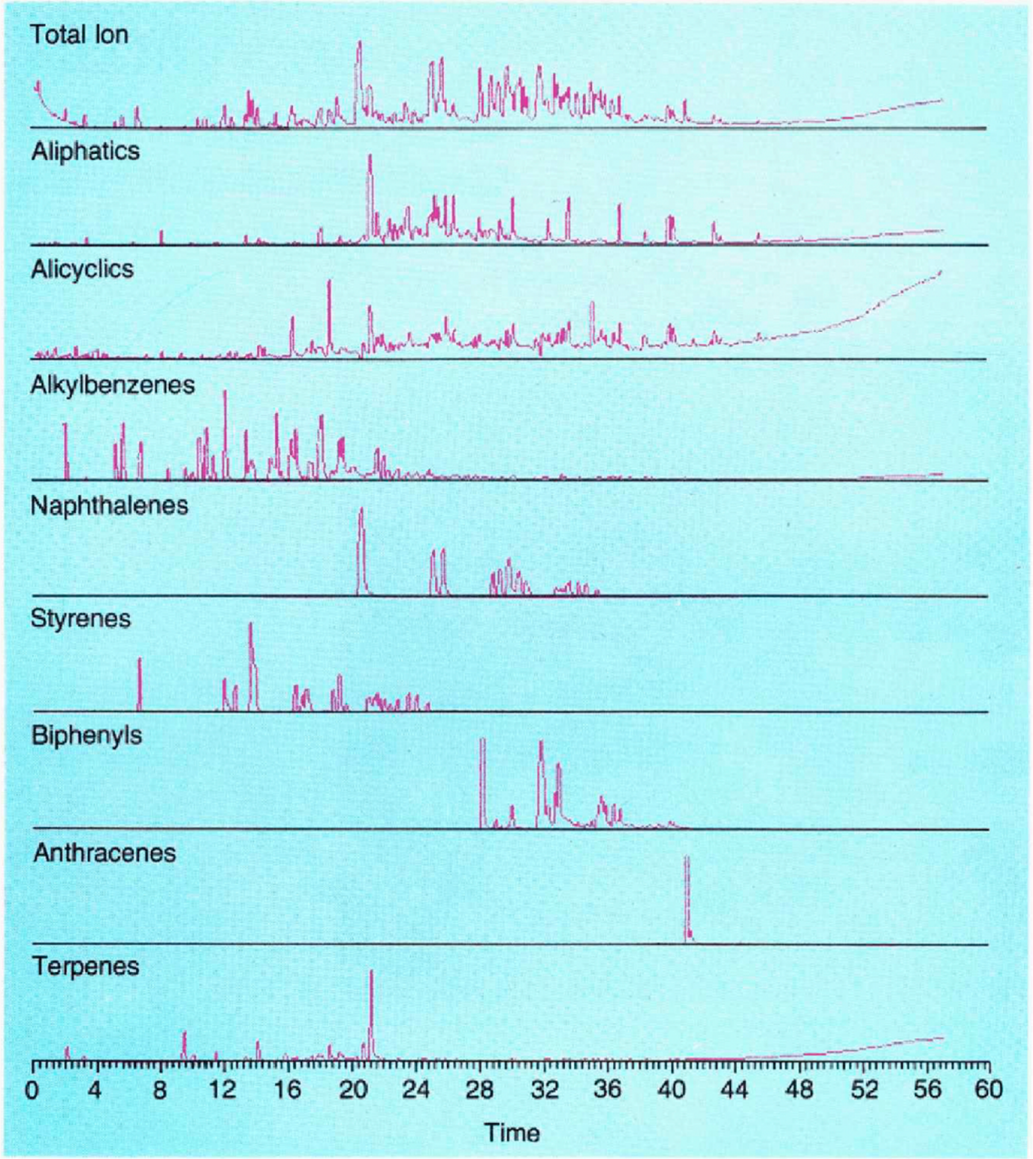
R. M. Smith’s casework sample of residues in a crack in concrete flooring from a structure fire in 1982. Reproduced from ref (102). Copyright 1982 American Chemical Society.

In 1970, R. A. Hites and K. Biemann showed that homologous series of substances, like hydrocarbons, could be readily observed by monitoring specific ions or groups of ions as a function of retention time.^100^ The technique was known as mass chromatography for more than a decade before the term extracted ion chromatography (EIC) took root. In 1977, M. H. Mach was arguably the first to apply GC-MS to ignitable liquid residues in a forensic context, but his gasoline samples were evaporated to extremely high levels (all> 95% weathered), so the findings are not very relevant to casework, where, anecdotally, gasoline is typically weathered between 50 and 80%.^101^ In the early 1980s, R. M. Smith described the application of mass chromatography to the GC-MS analysis of ignitable liquid residues (Figure 5).^102,103^

His table of selected fragments to help identify different classes of hydrocarbons in different distillates (Table 2) became the method of choice for practitioners, and it served as the foundation for the first consensus standard on the topic in 1997, called ASTM 1618-97.^104^

**Table 2:**
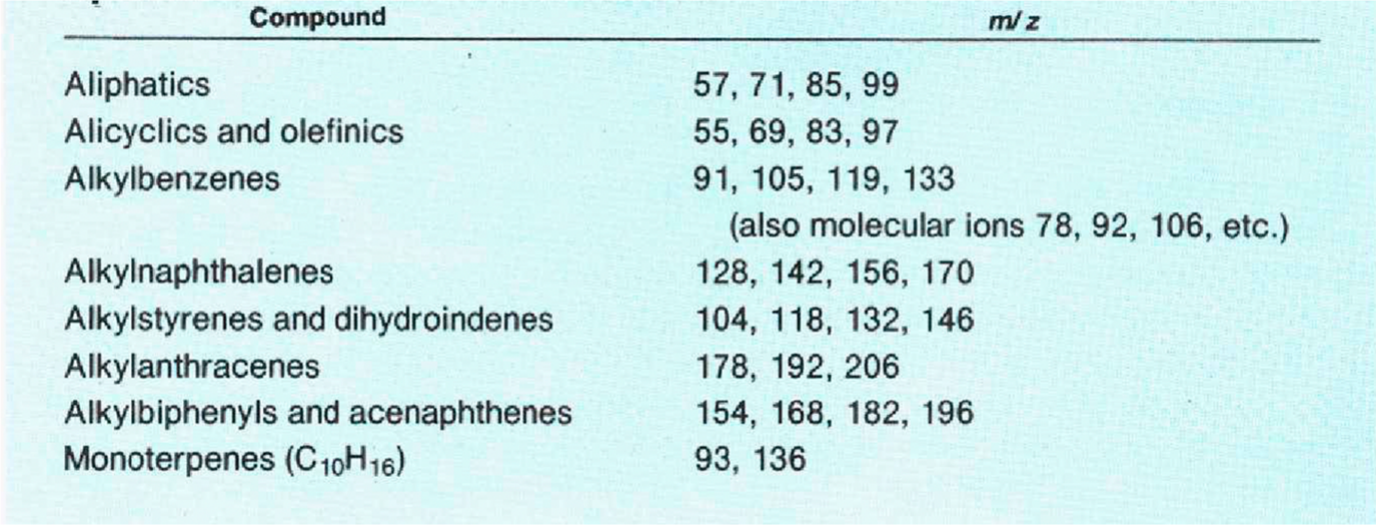
Representative Ions Normally Present in Mass Spectra of Common Accelerants^a^

a This table began in ASTM D 2789-69, a test method for gasoline, and evolved into a table seen today in ASTM E1618-19, a test method for ignitable liquids. Reproduced from ref (102). Copyright 1982 American Chemical Society.

In 1984, R. L. Kelly and R. M. Martz of the FBI reported a similar table to identify ignitable liquids in fire debris,^105^ which they said was adapted from ASTM D2789: Standard Test Method for Hydrocarbon Types in Low Olefinic Gasoline by Mass Spectrometry. ASTM D2789 was first approved in 1969 but was withdrawn as a standard in 2023, so all traces of the legacy documents have been removed from the ASTM Web site and only archived versions are available outside of ASTM.

Those knowledgeable about the origins of commercial mass spectrometers will not be surprised that the mass spectrometry community was already well-positioned to tackle the interpretation of data from hydrocarbon mixtures; In 1937, Herbert Hoover, Jr. had formed the Consolidated Engineering Company (CEC) specifically for the purpose of producing mass spectrometers to assist the US with prospecting petroleum deposits.^106^ In 1942, CEC had installed the first CEC 21–101 mass spectrometer at the Atlantic Refining Company of Philadelphia, and Washburn et al. published its first of many applications to hydrocarbon mixtures the following year.^107^

In the late 1980s and early 1990s, additional studies from W. Bertch et al.^108−110^ and R. O. Keto et al.^111−113^ helped support some key principles in the inaugural edition of ASTM E1618 in 1997. Essentially, the founding principles were: (1) that different types of ignitable liquids contained different subclasses of compounds that have characteristic fragments based on structural relationships; (2) that subclasses and homologous series show conserved characteristic patterns within different classes of ignitable liquids; (3) that the signal-to-noise ratios for the characteristic patterns of compound subclasses can be greatly enhanced through extracted ion monitoring; and (4) that pyrolysis of organic materials often forms the same characteristic hydrocarbons but in different/random relative abundances.^114−118^ Since 1997, ASTM E1618 has been updated about every five years, and it currently serves as a gold standard in the analysis of ignitable liquids in fire debris.^119^ The NIST Organization for Scientific Area Committees (OSAC) has a subcommittee on Ignitable Liquids, Explosives, and Gunshot Residue that has recently drafted a replacement standard for ASTM E1618, but the same fundamental principles remain.^120^

In 1997, D. A. Sutherland reported on a case in which he used GC-MS/MS to help improve the signal-to-noise ratio for the volatile components in a soil sample taken from the basement of a burned-down home.^121^ The gasoline residues were not observable using conventional GC-MS but were readily apparent using GC-MS/MS. Despite the obvious advantages of tandem MS, GC-MS/MS is not a well-established technique in crime lab settings. In contrast, LC-MS/MS has been the workhorse of forensic toxicology since the development of commercial instruments in the 1990s.^122−124^

## Gunshot Residue (GSR) and Explosives

Gunshot residue (GSR) has had several monikers, including cartridge discharge residue (CDR) and firearm discharge residue (FDR).^125,126^ GSR comprises the original and degraded particles from the primer and the propellant. Preceding the 1950s, one of the most common tests to determine whether or not someone had fired a gun by detecting GSR on their hands was the paraffin test, which involved pouring hot paraffin wax over a suspect’s hand and conducting a color test on the cooled, lifted wax.^127^ More than 30 years after its accepted use in *Commonwealth v. Westwood* in 1936, the paraffin test finally underwent some validation studies, which it promptly failed.^128^ The validation studies showed that the test was highly susceptible to false positives, including rust, fingernail polish, soap, and even tap water.^129−131^ The test was finally dropped, and forensic scientists developed alternative elemental and mass spectrometric approaches to identifying GSR, including neutron activation analysis (NAA),^132^ graphite furnace atomic absorption spectroscopy (GFAAS),^132^ GC-MS,^133^ inductively coupled plasma-MS (ICP-MS),^134^ liquid chromatography-tandem mass spectrometry (LC-MS/MS),^135^ and most recently desorption electrospray ionization-tandem mass spectrometry (DESI MS/MS).^136,137^

### A Smoking Gun

In 1982, *Dowland v. Lyman Products for Shooters*, a gun owner tried to sue a gun manufacturer for injuries after it exploded in his hands when he fired it.^138^ The Supreme Court of Utah found the application of GC-MS to be acceptable for resolving smokeless powder from black powder. The Court therefore found that he had intentionally used the wrong ammunition, so the gun was not defective. In 1994, the American Society for Testing and Materials developed a standard (ASTM E1588) that recommended scanning electron microscopy/energy dispersion X-ray spectrometry (SEM-EDS) to determine the presence of lead, antimony, and barium in the appropriate morphological particles, and SEM-EDS remains the method of choice for today’s analyses of GSR.^139^

In 1981, in his first of many books on the topic, J. Yinon explained how the extreme sensitivity and selectivity offered by mass spectrometry makes it an ideal tool for the identification and forensic analysis of high explosives.^140^ However, certain explosives are obviously fragile, so they tend to decompose in hot/dirty injection ports and give weak or no molecular ions by EI-MS.^141,142^ To gain selectivity and improve detection limits, much of the early work in the 1970s therefore focused on chemical ionization.^143−145^ Although mass spectrometers had been used for real-time atmospheric sampling of explosives since the 1970s, the National Research Council has only recently identified mass spectrometry as a desirable replacement for the cheaper, but poorer resolution, ion mobility analyzers for use at security checkpoints and border crossings.^140,146^

### Stable Isotopes Provide Investigative Leads

A more recent mass spectrometric capability in the geographic provenancing of explosives is using IRMS.^147−153^ IRMS now meets Daubert criteria for admissibility, and many reviews describe the huge variety of forensic applications of IRMS.^154−160^ IRMS has also been used in specific cases to help provide investigative leads for the identification of John and Jane Does.^159,161^

### Not Always a Silver Bullet

The first successful attempt to characterize bullets using mass spectrometry was by FBI analysts in 1975.^162^ Haney and Gallagher used spark source mass spectrometry (SSMS) to assess the abundance of about a dozen elements in different bullets, and they showed both intrabox and interbox variability within a brand and much larger interbox variability between brands.^162^ However, the technique does not appear to have caught on with practitioners. Starting around 1980, the FBI used comparative bullet lead analysis (CBLA) for more than 20 years and in more than 2,400 cases before a court found that the lab’s analysts had been miss-interpreting the results the whole time.^163^ In *Ragland v. Kentucky*, the Supreme Court of Kentucky ruled CBLA by ICP atomic emission spectroscopy (AES) to be inadmissible, which, of course, caused chaos with all the cases in which CBLA had been used.^164^ After the introduction of ICP-MS by R. S. Houk et al. in 1980, commercial instruments became a mainstay for trace metals analysis in just about every industry except the forensic community.^165^ One can only assume that the previous problems with ICP-OES for CBLA meant that there was little enthusiasm to stoke the fire by introducing ICP-MS for the same application, despite its superior figures of merit to ICP-OES.^166^ Although ICP-MS never caught on for metals analysis in forensic laboratories, it certainly did for glass, as described below.

## Trace, Fibers, and Hair

Most inorganic elements are only present as trace, incidental impurities in human hair, so early applications of mass spectrometry to human hair focused on the detection of the most abundant elements. In the 1930s, many scientists evaluated the levels of iron in human hair relative to different traits using chemical extraction and wet chemical techniques.^167−178^ In the 1950s and early 1960s, spectroscopic methods like flame atomic absorption (FAA) enabled the detection of the most abundant metals like iron and copper and even mercury and lead exposure in cases of poisoning.^179−184^ By the 1960s, neutron activation analysis (NAA) achieved new levels of detection for the few elements that were amenable to NAA.^185^ In 1969, J. P. Yurachek et al. analyzed 22 elements in human hair in a single analysis using SSMS.^184^ However, spark sources typically struggled with stability and reproducibility, and stories about unintended discharges to human hands during maintenance helped prevent SSMS from reaching mainstream in forensic laboratories.

### Splitting Hairs

Ion microprobe mass spectrometry (IMSS), now called secondary ion mass spectrometry (SIMS), was first presented by R. Castaing and G. Slodzian 1962.^186,187^ In its first forensic application in 1977, researchers at the McCrone Institute used IMMS in *United States v. Brown* to link a suspect’s hair with those found at a fire-bombed Planned Parenthood clinic.^188^ There was much debate in court about the validity and application of IMMS for human hair because it had never been applied for this purpose before. After much legal wrangling, IMSS as a technique was found to be reliable, but it is application to human hair ultimately failed to meet the admissibility criteria of the day “because the analytical technique used had not attained general acceptance in the scientific community, nor were the experiments conducted shown to be sufficiently reliable and accurate.”^188^

### Without a Trace...of Fingerprints

In the fall of 1998, a three-year-old girl was abducted and brutally murdered. Witnesses placed the girl in the suspect’s car, but investigators could not find any fingerprints from the child. That fall, M. Buchanan and co-workers used GC-MS to show that the chemical composition of the fingerprints of children were very different from those of adults, and that the lack of squalene and heavy lipids in the children’s fingerprints meant that, unlike those of adults, their fingerprints typically disappeared within 24 h.^189^ GC-MS therefore could not detect any traces of the girl’s fingerprints.

### With a Trace...of Plastic

**Figure 6:**
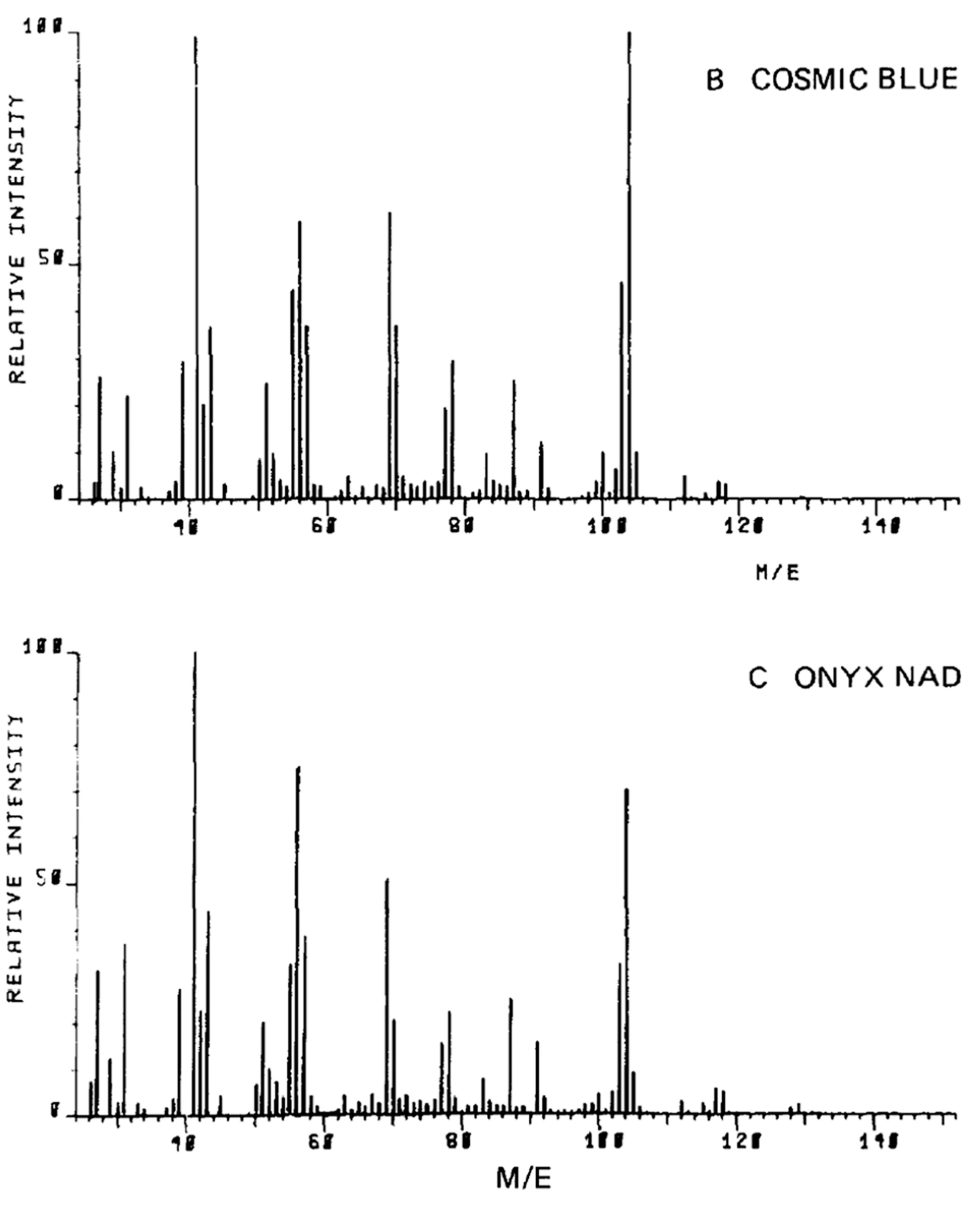
Examples of mass pyrograms of different colored acrylic car paints. Reproduced with permission from ref (190). Copyright 1977 Elsevier.

Pyrolysis-GC-MS (Pyr-GC-MS) was introduced to the forensic community by J. A. Zoro and K. Hadley in 1976 when they described a case where they linked an antioxidant in the trace fragments of a polymer in blades of a hacksaw to those of a stolen polymer-coated cable.^12^ R. Saferstein et al. and J. C. Hughes et al. widened the opportunities for Pyr-GC-Ms in their 1977 studies on man-made fibers and polymers, including car paint (Figure 6).^190,191^

After these first demonstrations, Pyr-GC-MS was commonly used in crime laboratories to examine a wide range of trace materials, including binders, tapes, polymers and various components of automotive or architectural paints. In fact, such applications have been recommended by the support working group on materials analysis (SWGMAT) for more than 20 years.^192^ Methods such as LA-ICP-MS for trace metals in automotive paints and IRMS for the analysis of white architectural paints have been applied with success in research settings, but do not appear to have been tested in court.^193−195^ Pyr-GC-MS is still commonly used in today’s trace laboratories to study synthetic fibers and polymers.^196^

### Glass Analysis

In 1978, J. Locke et al. demonstrated that SSMS could discriminate between small glass samples, but large, expensive double sector instruments were required to cope with the wide energy spread of the generated ions, so the method was never practical for crime laboratories.^197^ After R. S. Houck successfully coupled mass spectrometry to ICP in 1980,^165^ the forensic community demonstrated that the intra- and intervariability of different elements in glass were sufficiently different to enable ICP-MS to be applied to glass samples in forensic contexts.^198−200^ In 2002, J. R. Almirall’s group began using laser ablation (LA) as an introductory method for ICP-MS for glass, paint, soil, and metals, and in 2003, his group showed that ICP-MS could confidently associate glass fragments collected from a suspect in a case with glass fragments from different car windows that he had broken.^201−204^ In 2004, the first ASTM standard appeared.^205^ By 2005, LA-ICP-MS had been validated in several laboratories and was demonstrated to be reliable for interlaboratory comparisons of trace glass samples.^206,207^ An ASTM method for LA-ICP-MS of glass soon followed, and the community is still thriving today.^208^ Numerous interlaboratory databases are continually being updated to help practitioners determine weights of evidence when using ICP-MS or LA-ICP-MS on glass.^209,210^

### A Look to the Future

In addition to understanding the past, historical perspectives can provide some context with which to better accept the present and pontificate about the future. Obviously, the performance characteristics of mass spectrometers and hyphenated techniques will continue to improve, and we can expect to see gains in resolving power, limits of detection and mass spectral identification algorithms. Less obvious are the long-term prospects of forensic mass spectrometry. Two current and major trends in mass spectrometry are the development of fieldable instruments and ambient ionization methods, which aim to reduce the extent of sample preparation necessary for analysis to enable real-time data acquisition and real-time decision making. As a reminder, first examples of field-deployable mass spectrometers began in the late 1940s by Nier’s group, so they are not new concepts. These topics are exquisitely covered in a recent review by Evans-Nguyen et al.^211^ Fieldable mass spectrometers are already actively employed in the criminal justice systems of other countries, and the success of such programs will likely drive other countries to adopt such protocols.^212^

On a more philosophical note, one of the more profound possibilities in forensic science relates to a complete mass-spectrometric molecular inventory of human habitats, as promulgated by Dorrestein’s group at UCSD.^213^ In perhaps the most true-to-form example of Locard’s exchange principle, Dorrestein’s group is currently using high resolution LC-MS to conduct thorough molecular inventories of human surfaces (e.g., skin) and the surfaces with which we exchange microbes, skin cells, lipids, and metabolites, among others. The possibilities of 3-dimensional molecular cartography in human environments seem endless, and there will be a vast number of scenarios and factors that need to be assessed before such capabilities could be used in court to convict or exonerate a suspect of a potential crime.

## Conclusions

Mass spectrometry has experienced a privileged position in the forensic community,^9,214,215^ with past and present legal critiques agreeing that mass spectrometry is “the near universal test for identifying unknown substances” and a gold standard of instrumental analysis.^216^ Still, the mass spectrometry community recognizes that even gold standards can have their limitations and that any application of mass spectrometry to a particular problem must be fit for purpose.^217−220^ Toward this end, consensus-based standards, like ASTM methods, help recommend best practices for how to apply mass spectrometry to different problems in forensic science.

Whereas researchers at federal laboratories and universities continue to develop new and promising applications with cutting-edge instruments, crime laboratories often struggle to secure the finances and time to obtain and validate them. Sometimes, practitioners also lack awareness of the latest developments in instrumentation or applications because their laboratories do not have the finances or time to send them to conferences and workshops for appropriate continuing education. These issues have been repeatedly identified since the 1950s, and they are equally relevant today.^13,216,221^ Still, mass spectrometry remains one of the more reliable forms of scientific evidence, and it either is not mentioned or receives praise from even the harshest critiques of the forensic sciences, including the landmark 2009 report by the National Academy of Sciences (NAS) and the 2016 President’s Council of Advisors on Science and Technology (PCAST) report.^130,216,222^ Hopefully, if scientists continue to apply mass spectrometry in dependable and fit-for-purpose ways, they should safely avoid the problems encountered in *US v. 2,116 Boxes of Boned Beef* in which GC-MS experts were “disregarded as being of any scientific assistance to the Court.”

## Supporting Information Available

Poster version of this review and a visual timeline (PDF)
