# Supplementary material for: Forensic Mass Spectrometry: Scientific and Legal Precedents
Source: J Am Soc Mass Spectrom. 2023 Jun 5;34(7):1210–24. doi: 10.1021/jasms.3c00124 (PMC10326920; doi:10.1021/jasms.3c00124)
Supplement: Supplementary file 1 — js3c00124_si_001.pdf [file js3c00124_si_001.pdf]

Supporting Information

Forensic Mass Spectrometry: Scientific and Legal Precedents

Glen P. Jackson[1,2*] and Mark A. Barkett[3]

[1]Department of Forensic and Investigative Science, West Virginia University, Morgantown, WV 26506-6121

[2]C. Eugene Bennett Department of Chemistry, West Virginia University, Morgantown, WV 26506

[3]Dover Chemical Company, Dover, OH 44622

*glen.jackson@mail.wvu.edu; 304-293-9236

Includes two posters and a list of references presented at the 2020 ASMS Conference on Mass Spectrometry and Allied Topics (Virtual due to COVID).

History of Forensic Mass Spectrometry

ASMS History Committee Poster, 2020
Glen P. Jackson[1]* and Mark A. Barkett[2]
[1]Department of Forensic and Investigative Science, West Virginia University
[2]Dover Chemical Corporation

Introduction

Mass spectrometry (MS) has long held respect in the forensic community,[1-3] and contemporary legal critiques of forensic science agree that MS is "the near universal test for identifying unknown substances" and one of the gold standards of instrumental analysis.[4] Still, the MS community recognizes that even 'gold standards' have their limitations and must be fit for purpose.[5-8] In the history of forensic MS, one of the first mass spectrometers in a forensic laboratory was in Birmingham, UK in 1973. Zoro and Hadley reported details of the workload of this first mass spectrometer in a fascinating summary in 1976 (see **Table 1**). The greatest proportion of cases involved the analysis of drugs, both in bulk form and in human bodily fluids. Fast forward 55 years to the modern era, and according to the Office of Justice Statistics, drug identifications remain the most frequently submitted evidence request in a typical forensic laboratory.[9]

Number of cases	Type of case
59	Illegal possession of drugs
47	Suspicious death
18	Explosives
17	Arson
10	Miscellaneous
8	Administration of noxious substance
7	Driving under the influence of drugs
7	Malicious damage
4	Documents
2	Biology

Table 1. Distribution of case types of the first year of operation (1973) of a mass spectrometer in the Home Office Central Research Establishment in Birmingham, UK.[8]

Drugs and Toxicology

As described above, MS has found widest application in the analysis of drugs, drug metabolites and drug paraphernalia. In 1929, Bleakney developed the electron ionization (née impact) source for the analysis of gases and inorganic vapors.[10] He then extended the work to simple organics, like methane, in the late 1930s.[11] Although mass spectrometers were commercially available in the mid-1940s, they were big, expensive, oftentimes customized and difficult to operate.[12] Also, there was no guidance for interpreting the complicated spectra. Defying this latter problem, Beynon and McLafferty each contributed to the launch of organic MS in the mid-1950s.[13-16] From 1959 onwards, Biemann and Djerassi's groups were prolific in extending MS's capabilities to the analysis of natural products and botanical extracts, including various alkaloids, cannabis and cocaine.[17-22] At the Food and Drug Administration (FDA), in 1968, Martin and Alexander explained how they used high resolution MS (HRMS) and "cracking patterns" to help identify the hallucinogen dimethyltryptamine (DMT) in a casework sample.[23] They reported that "a problem that would have constituted a major research project a few years ago was reduced to an exercise problem in spectroscopic identification".

In 1968, Coutts and Locock reported on the characterization of eight common barbiturates and their mixtures in pills and capsules.[24] Between 1968-1970, Bellman and coworkers at the FDA reported on the analysis of several hallucinogenic drugs using MS.[23, 25, 26] These early applications included lysergic acid diethylamide (LSD), mescaline, psilocin and psilocybin, among others. Others quickly followed suit.[27, 28]

In 1970, Althaus et al. reported on the use of a gas chromatography-MS (GC-MS) system at Massachusetts Institute of Technology (MIT) to detect Darvon in stomach contents to solve a drug overdose case.[29] The case was apparently solved in about a day, which is a far cry from today's backlogs but admittedly took a team of MIT MS experts to complete. By 1971, Fales' group at the National Institutes of Health (NIH) had solved more than 100 overdose cases using GC-MS and computer-assisted database searching,[30] including the analyses of blood serum and stomach contents. In 1972, Skinner et al. reviewed the status of GC-MS for forensic toxicology.[31] In the same year, Green showed the potential of MS "fragmentography" to identify drugs from the headspace of drug samples, in pseudo real-time, with no sample preparation (besides dissolution in acid or base) (**Figure 1**).[32] He even showed the ability to detect alcohol in circulating blood *in vivo* (**Figure 1**)! Of course, the field of ambient sampling MS[33] has recently witnessed an enormous resurgence following the introduction of desorption electrospray ionization (DESI)[34] and direct analysis in real time (DART)[35] in the early 2000s.

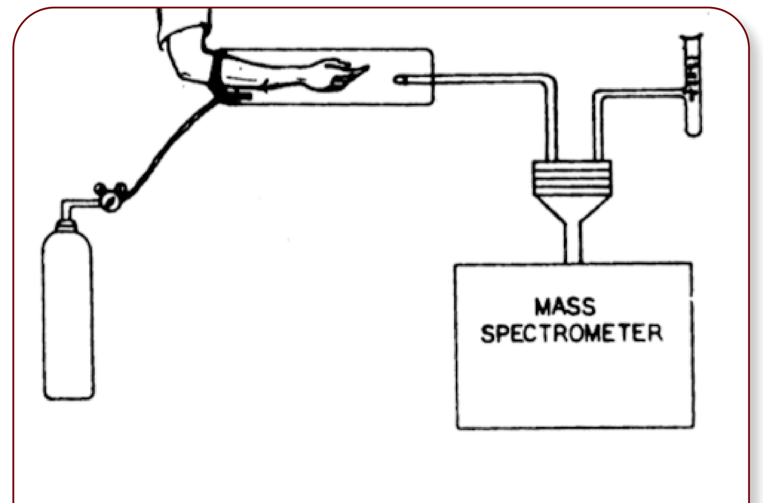

Figure 1. Schematic representation of the setup used by Green in 1972 for real-time drug monitoring and ambient sampling of drugs of abuse. Reproduced from reference.[32]

In 1973, a Swedish team developed a GC-MS assay for Δ^9-Tetrahydrocannabinol (THC) in human blood that was sensitive enough to detect if someone had smoked "one half-billionth of a gram".[36] The same year, Saferstein and Chao reported on the use of chemical ionization (CI)—which had been introduced by Munson and Field in 1966[37]—to analyze drugs and drug mixtures.[38] By 1974, Jardine and Fenselau added charge exchange ionization to the analysis of drugs (**Figure 2**),[39] and many other groups were adding to the "forensic mass spectrometry" literature. Many early forensic applications were captured in Fenselau's comprehensive review of GC-MS in 1974.[40]

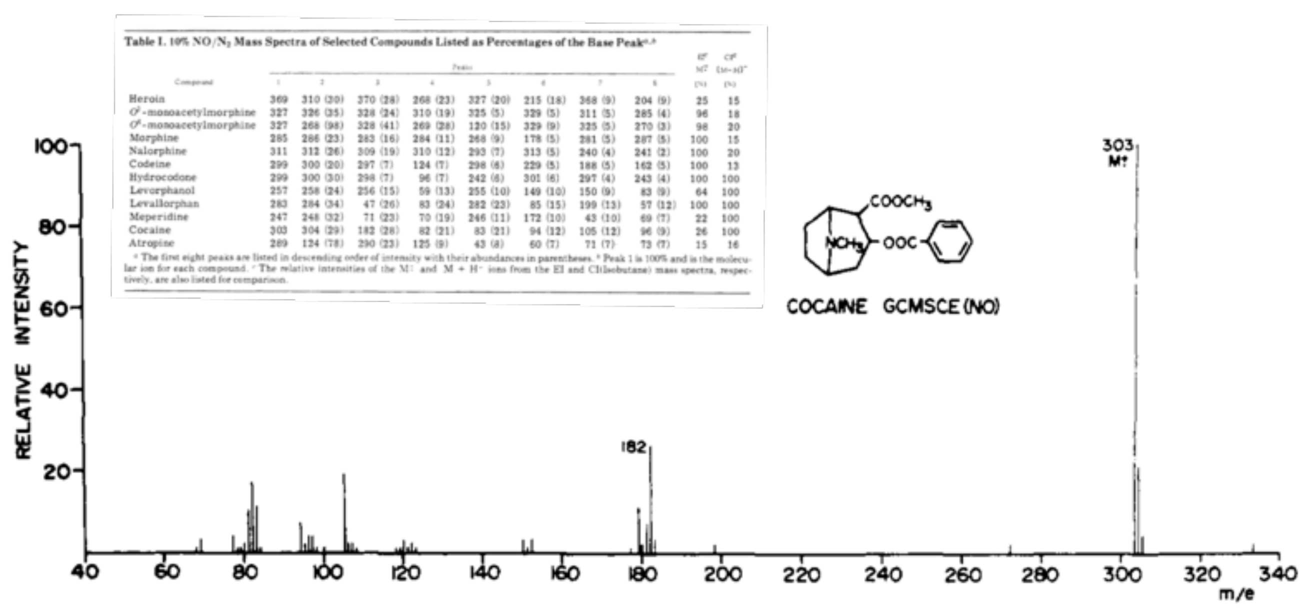

Figure 2. In 1974, Jardine and Fenselau compared electron ionization, chemical ionization and charge exchange ionization of a selected range of morphine and tropane alkaloids.[39]

In 1977, MS data from the Environmental Protection Agency (EPA) was admitted as evidence in a case involving the detection of a pesticide known as tetrachlorodibenzo-*p*-dioxin (TCDD) in animal tissues from the Siuslaw National Forest.[41] The following year, a judge ruled to allow the MS test results as evidence in a capital murder case.[42] In 1978, GC-MS results were also admitted in a high priority case involving the American Meat Institute.[43] The concern was whether or not bacon had been adulterated by the addition of elevated levels of nitrosamines. MS was used in this case because it "is widely regarded as the best available technology".[43]

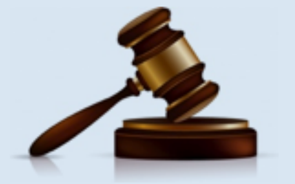

Designer Law for Designer Drugs: In 1986, the Controlled Substance Analogue Enforcement Act (CSAEA, 21 U.S. C. § 813) was signed into law to control compounds that were "substantially similar" to drugs that were scheduled in the Controlled Substances Act (CSA) of 1970 (Pub. L. No. 91-513, § 202, 84 Stat. 1236). In 1992, *United States v. Forbes* (806 F. Supp. 232), a judge ruled that CSAEA's language was "unconstitutionally vague" and that alpha-ethyltryptamine (AET) was not substantially similar to the scheduled drugs dimethyltryptamine (DMT) or diethyltryptamine (DET). Instead of modifying the language, the DEA simply added AET to the list of scheduled drugs. In *US v. Washam* (2002, 312 F.3d 926), another judge found the language in CSAEA to be valid. Additional cases since 2003 have narrowed the language to include the "same core arrangement of atoms" test or the "structure and effect" test (e.g. *US v. Klecker*, 2003, 228 F. Supp. 2d 720).

In the 1970s, a ruling was overturned thanks to the ability of negative CI-MS to detect dibromopropanol—a metabolite of the flame retardant Tris(2,3-dibromopropyl) phosphate (Tris-BP)—in childrens' urine.[44, 45] In a review article on urinalysis in 1979, a probation officer named Bigger stated that although MS was "the most sensitive and specific technique available", it was "too expensive and too slow to be commonplace".[46] A survey of 100 crime labs in 1973 showed that mass spectrometers were the most desired piece of equipment, if unlimited funds were available,[47] and it only took a few years before GC-MS was commonplace in forensic laboratories.

Although Djerassi and others had analyzed various tropane alkaloids using MS in the 1960s and early 1970s,[18, 48-50] more than a dozen groups had contributed to the analysis of cocaine and its metabolites by the end of the 1970s.[30, 38, 39, 50-53] However, Kondrat and Cooks were arguably the first to perform tandem mass analysis on cocaine in 1978.[54]

After the Controlled Substances Act of 1970, arguments continued over the need to distinguish harmless *d*-cocaine from the active drug, *l*-cocaine.[55-58] After some embarrassing problems with testimony about cocaine isomers,[59] one expert noted that *d*-cocaine "had also never been seen apart from *l*-cocaine".[60] Thanks to his testimony, we no longer have to identify the isomers of cocaine!

At the 1972 Olympics in Munich, GC-MS screening produced 7 adverse findings from 2079 tests of athletes' blood and urine.[61, 62] The turnaround time averaged less than 24 hours per sample! Another major milestone was established in 1994 when Becchi et al. validated a method to distinguish exogenous from endogenous testosterone using GC-combustion-isotope ratio mass spectrometry (GC-IRMS).[63]

Arson

In 1959, Joseph Nicol, a firearms technician at the Chicago police crime lab, suggested that crime labs at large universities or oil companies could run GC-MS tests for high priority arson cases.[64] In 1976, Zoro and Hadley[65] published a review article that outlined the use of GC-MS for ignitable liquids (née accelerants) in place of GC-flame ionization detection (GC-FID).[66, 67] **Figure 3,** from Zoro and Hadley's article, shows chromatograms of headspace samples of a suspected ignitable liquid and of the fire debris, the latter of which showed three additional compounds relative to the ignitable liquid.[68]

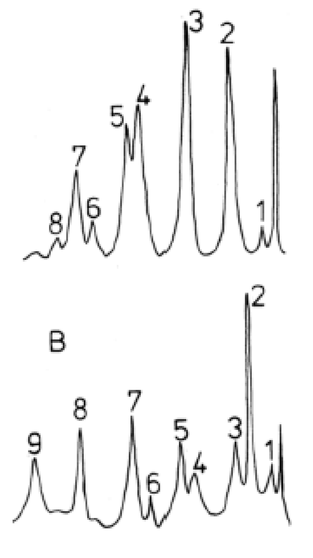

TABLE 2
ANALYSIS OF HEADSPACE SAMPLES*

Peak	Accelerant Identification	Peak	Debris Identification
		1	carbon disulphide
1	? cyclopentadiene		
		2	? acetone
2	benzene	3	benzene
3	toluene	4	toluene
		5	? octene
4	xylene	6	xylene
5	xylene and styrene	7	styrene
6	? methylethylbenzene		
7	? propyl and propenyl benzene	8	? propyl and propenyl benzene
8	indene		
		9	unknown

Figure 3. GC-MS results of headspace samples from Zoro and Hadley. Top=suspected ignitable liquid, bottom=fire debris, which contains the same compounds in different abundances plus three additional peaks.[68]

Gunshot Residue (GSR) and Explosives

The composition of gunshot residue (GSR), also known as cartridge discharge residue[69] (CDR) or firearm discharge residue[70] (FDR), consists of the consumed and unconsumed particles from the primer and the propellant. The first tests used to determine whether or not someone had fired a gun by GSR was the paraffin test, which involved pouring hot paraffin wax—often painfully!—over a suspect's hand and conducting a color test on the cooled wax.[71] Because of the general unreliability of the color tests,[72-75] alternative elemental and mass spectrometric approaches were developed, including neutron activation analysis (NAA)[76], graphite furnace atomic absorption spectroscopy (GFAAS),[76] GC-MS,[77] inductively coupled plasma-MS (ICP-MS),[78] liquid chromatography-tandem mass spectrometry (LC-MS/MS),[79] and DESI MS/MS.[80, 81] Whereas it was acceptable to use GC-MS in GSR cases in the 1980's,[82] the American Society for Testing and Materials (ASTM E1588-17) developed a standard in 1994 that recommended scanning electron microscopy/energy dispersive x-ray spectrosopy (SEM-EDS) to determine the presence of lead, antimony, and barium in the appropriate morphological particles; SEM-EDS remains the method of choice for GSR.[83]

As explained by Yinon in 1981—in his first of many books on the topic—the extreme sensitivity and selectivity offered by the mass spectrometer make it an ideal tool for the identification and forensic analysis of chemical explosives.[84] However, many explosives give no molecular ion by EI-MS, so much of the early work in the 1970s focused on chemical ionization.[85-87] Although mass spectrometers have been used as explosive detectors or "sniffers" since the 1970s,[84] they have only recently been identified by the National Research Council as desirable replacements for the considerably cheaper, but poorer resolution, ion mobility analyzers.[88]

A newer application to the field of forensics is the discrimination of explosives by using IRMS.[89-95] IRMS now meets Daubert criteria for admissibility,[96] and several reviews describe the huge variety of forensic applications of IRMS.[97, 98]

Comparative Bullet-Lead Analysis (CBLA)

The first successful attempt to match bullets with MS was in 1975 by Haney and Gallagher using spark source mass spectrometry (SSMS).[99] After the introduction of ICP-MS in 1980,[100] commercial instruments became a mainstay for trace metals analysis in just about every industry except the forensic community.

Because of problems with reporting the evidentiary value of CBLA, a court ruled CBLA by ICP-atomic emission spectroscopy (AES) to be inadmissible.[101] Although ICP-MS[102] and laser ablation (LA)-ICP-MS have the power to analyze sub-ppb level impurities, and even obtain isotopic analyses of trace elements, very few crime labs can afford the expense of these techniques.

Trace, Fibers & Hair

Early applications of MS for hair analysis focused on the detection of trace-level inorganic impurities. Due to the low concentration of inorganic elements in human hair, only the most abundant elements could be studied. As early as the 1930s, scientists studied the concentration of iron in human hair using chemical extraction.[103-114] From the 1950s through the early 1960s, spectroscopic methods like flame atomic absorption (FAA) enabled the detection of the most abundant metals like iron and copper[115-117] and even mercury and lead exposure in cases of poisoning.[118-120] By the 1960s, NAA achieved new levels of detection for a few elements that were amenable to NAA.[121] In 1969, Yurachek *et al*. performed the first direct mass spectrometric study of trace elements in human hair using SSMS.[120]

Ion microprobe mass spectrometry (IMSS)—now secondary ion mass spectrometry (SIMS)—was first presented by Castaing and Slodzian 1962.[122,123] In its first forensic application, researchers at the McCrone Institute in 1977 used IMMS to link a strand of a suspect's hair with hairs found at a firebombed Planned Parenthood clinic. After several appeals, IMMS was found to be reliable, but its application to human hair ultimately failed to meet the admissibility criteria of the day "because the analytical technique used had not attained general acceptance in the scientific community, nor were the experiments conducted shown to be sufficiently reliable and accurate."[124]

In 1976, Zoro and Hadley described the application of pyrolysis mass spectrometry (Py-MS) to identify the presence of an antioxidant in the trace fragments of a polymer in a hacksaw to those of the stolen polymer-coated cable.[68] Pyrolysis-GC-MS (Pyr-GC-MS) was introduced to the forensic community by Saferstein et al. and Hughes et al. in their 1977 studies on man-made fibers and polymers.[125, 126] Since then, Pyr-GC-MS has also been applied to other trace polymeric materials, including binder, polymers and organic pigments in the automotive or architectural paints. In fact, such applications have been recommended by the support working group on materials analysis (SWGMAT) for more than 20 years.[127] Since the publication of these recommendations, methods such as LA-ICP-MS for trace metals in automotive paints[128] and IRMS for the analysis of white architectural paints have been applied with success, although not tested in court.[97, 129] Pyr-GC-MS is still commonly used in today's trace labs to study fibers and polymers.[130]

In 1978 Locke et al. demonstrated that SSMS could discriminate small glass samples,[131] and after Houck successfully coupled MS to ICP,[132] the forensic community slowly demonstrated the use of ICP-MS for the analysis of trace impurities in forensic glass samples.[133-135] By 2003, laser ablation (LA)-ICP-MS, which is now an ASTM standard,[136] was also demonstrated to be reliable for comparisons (trace) glass samples.[137]

Conclusion

Mass spectrometry has a long and essential history in the legal community and continues to provide some of the most reliable evidence in the forensic sciences. Although MS techniques have evolved dramatically over the years, there remains the perpetual problem of adequately supplying the finances and education required for forensic laboratories to possess and use the latest capabilities. These issues were identified in the 1950's[64] and 1970's,[138] and they are equally relevant today.[4]

Acronym Index

APCI	Atmospheric Pressure Chemical Ionization	IMMA	Ion Microprobe Mass Analyzer
CBLA	Comparative Buller Lead Analysis	NIH	National Institutes of Health
CI-MS	Chemical Ionization-Mass Spectrometry	ICP-MS	Inductively Coupled Plasma-Mass Spectrometry
CSA	Controlled Substances Act	IRMS	Isotope Ratio Mass Spectrometry
CSAEA	Controlled Substance Analogue Enforcement Act	LA-ICP-MS	Laser Ablation-ICP-MS
DART	Direct Analysis in Real Time	LSD	Lysergic Acid Diethylamide
DESI	Desorption Electrospray Ionization	NAA	Neutron Activation Analysis
EA-IRMS	Elemental Analyzer-Isotope Ratio Mass Spectrometry	PSAT	Pressure Sensitive Adhesive Tape
EI-MS	Electron Ionization-Mass Spectrometry	Py-GC-MS	Pyrolysis-GC-MS
EDS	Energy Dispersive X-Ray Spectrometry	Py-MS	Pyrolysis-Mass Spectrometry
EPA	Environmental Protection Agency	SSMS	Spark Source Mass Spectrometry
DMT	Dimethyltryptamine	SEM-EDS	Scanning Electron Microscopy/Energy Dispersive X-ray Spectroscopy
FDA	Food and Drug Administration		
GC-MS	Gas Chromatography-Mass Spectrometry	TCDD	2,3,7,8-tetrachlorodibenzo-p-dioxin
GC-MS/MS	Gas Chromatography-Tandem Mass Spectrometry	USDA	United States Department of Agriculture
GSR	Gunshot Residue		

History of Forensic Mass Spectrometry

ASMS History Committee Poster, 2020
Glen P. Jackson[1]* and Mark A. Barkett[2]
[1]Department of Forensic and Investigative Science, West Virginia University
[2]Dover Chemical Corporation

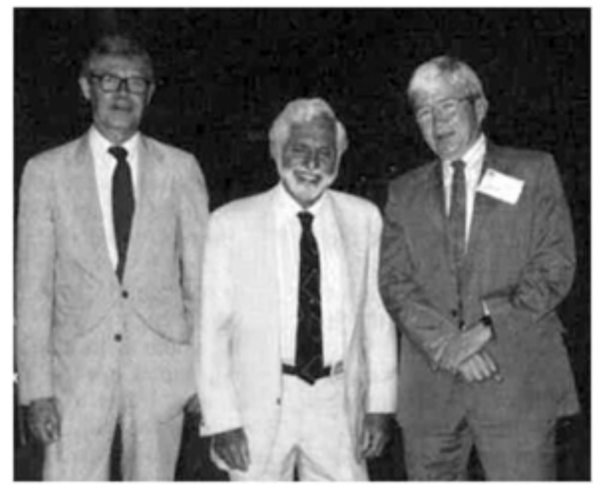

Pioneers of mass spectrometry, K. Biemann, C. Djerassi and F. L. McLafferty at the 38th ASMS Conference in 1990.

1959
K. Biemann's use EI-MS to distinguish the stereochemistry of cyclic alcohols in natural products, including steroids.[17]

1959
J. Nicol proposes the use of GC-MS for the analysis of ignitable liquid residues.[64]

1967
After ~30 years of use, Cowan and Purdon conduct first comprehensive validation of the paraffin color test for gunshot residue (GSR); found the test to be unspecific, but the test remains in practice for decades afterwards.[75]

1968
R. J. Martin and T. G. Alexander of the FDA publish on the use of MS "cracking patterns" to identify LSD, DMT and psilocybin in casework.[23]

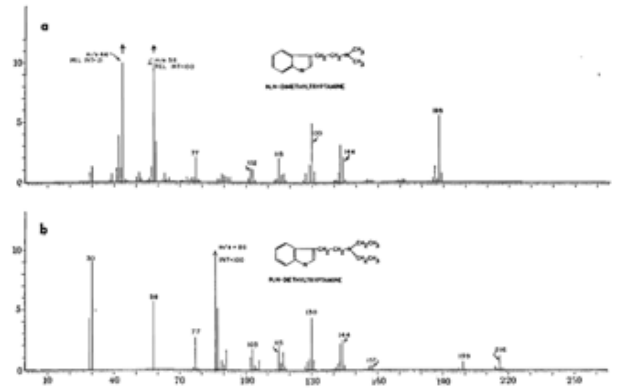

Fig. 2—Mass spectra of (a) N,N-dimethyltryptamine (DMT) and (b) N,N-diethyltryptamine (DET).

1970
H. M. Fales et al. apply CI-MS to barbiturates.[140]

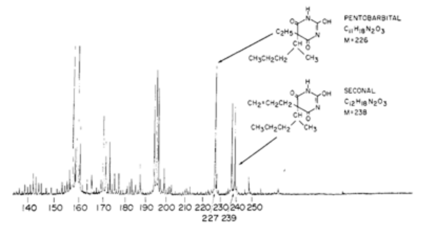

Figure 3. Chemical ionization mass spectrum of extract of gastric contents

1970
K. Biemann's group publish a series of manuscripts using MS and computer-assisted searches to identify drugs and drug metabolites in overdose cases, including Darvon in a comatose patient.[29]

1973
R. Saferstein and J. Chao use CI-MS to analyze 62 drugs.[38]

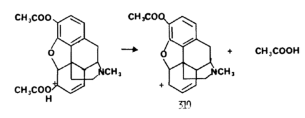

FIG. 3—Mechanism for the fragmentation of diacetylmorphine.

1973
A survey of US crime labs showed that 2 out of 37 respondents had GC-MS capabilities.[47]

1976
P. Bommer et al. first use isotope ratio mass spectrometry (IRMS) to distinguish between drugs with different origins; measured ^{2}H and ^{13}C ratios of different production batches of diazepam.[142]

1976
J. A. Zoro and K. Hadley publish a review of the use of MS in forensic laboratories in the UK; includes Pyrolysis-MS (Py-MS)of polymers and GC-MS of drugs and ignitable liquids.[68]

1977
R. Saferstein et al. and J. C. Hughes et al. publish separate accounts of Py-GC-MS of man-made fibers and polymers.[125, 126]

1977
In Citizens Against Toxic Sprays Inc. et al. vs USDA et al., the EPA uses low- and high-resolution MS to quantify the toxic herbicide TCDD in beef fat to levels of ~5 ppt.[41]

1980
GC-MS used to disqualify a horse from a trifecta race after detecting fentanyl in its urine.[143]

1981
In US vs Posey, GC-MS evidence upheld for cocaine because "d-cocaine has never been seen apart from l-cocaine;" analysts no longer have to confirm the isomeric form of cocaine.[60]

1993
Landmark case *Daubert vs Merrell Dow Pharmaceuticals* redefines federal rules of evidence for expert witness testimony.[144]

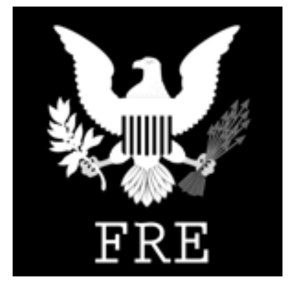

1999
Through a collaboration with J. R. Ehleringer et al., the DEA uses IRMS to determine the geolocation of illicit heroin and cocaine seizures.[146]

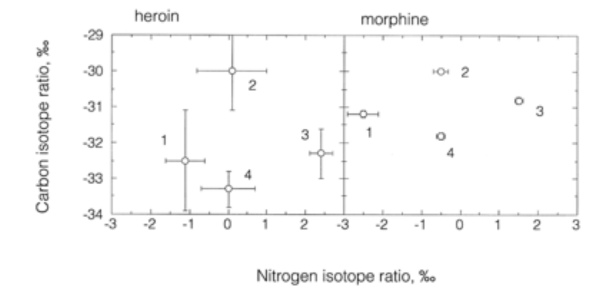

Fig. 1. Carbon and nitrogen isotope ratios of authenticated heroin (left plate) and associated morphine samples (right plate) originating from the major growing regions: Mexico (1), Southwest Asia (2), Southeast Asia (3), and South America (4). Error bars indicate 99% confidence interval.

2004
R. G. Cooks' group files invention disclosure and publishes on desorption electrospray ionization (DESI) MS for the ionization of polar samples, including drugs and explosives, on ambient surfaces.[34]

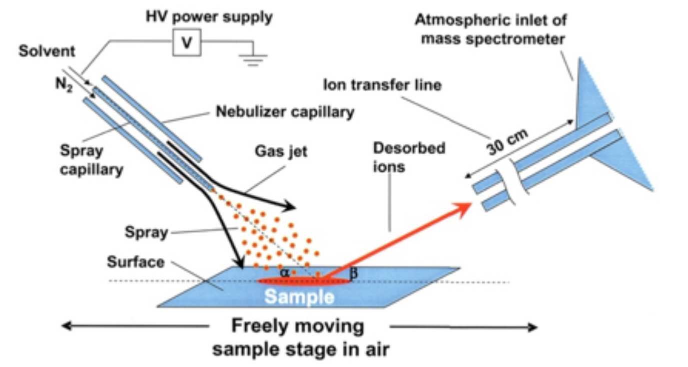

2008
W. Meier-Augenstein and I. Fraser use isotope ratio mass spectrometry (IRMS) to help identify the geographic origins and travel history of a mutilated murder victim.[147]

1964
C. Djerassi's group publishes dozens of articles describing the mass spectral analysis of natural products, including tropane alkaloids and cannabinoids.[18, 19]

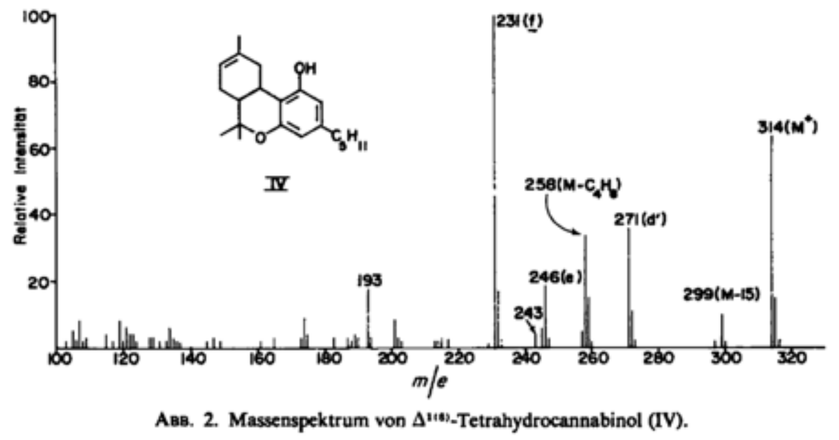

Abb. 2. Massenspektrum von Δ[1(6)]-Tetrahydrocannabinol (IV).

1965
R. A. Brown publishes chapter on "The mass spectrometry of hydrocarbons," including common fragments and mechanisms for aromatics, alkanes, alkenes, cycloalkanes and polyaromatics; lays the groundwork for ignitable liquid residue determinations.[139]

1968
R. T. Coutts and R. A. Locock analyze barbiturates from capsules and tablets using ether extractions and MS.[24]

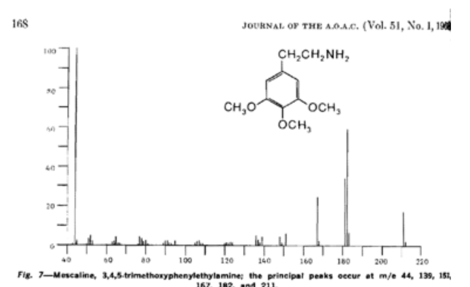

Fig. 7—Mescaline, 3,4,5-trimethoxyphenylethylamine; the principal peaks occur at m/e 44, 139, 151, 167, 182, and 211.

1968
S. W. Bellman and coworkers publish on the identification of novel hallucinogens in casework.[25, 26]

1971
The Federal Controlled Substance Act of 1970 becomes effective.[141]

1971
G. W. A. Milnes' group at NIH report on the use MS and an automated search algorithm to solve hundreds of overdose cases.[30]

1972
In the summer Olympics in Munich, GC-MS produces 7 adverse findings in 2079 blood and urine samples. The turnaround time was <24 hrs for each sample.[61, 62]

1972
D. E. Green describes the real-time, in-vivo analysis of alcohol and drugs from humans using ambient sampling and "mass fragmentography."[32]

1974
R. G. Gillis et al. and J. Yinon publish separate accounts of CI-MS to the analysis of high explosives.[85, 86]

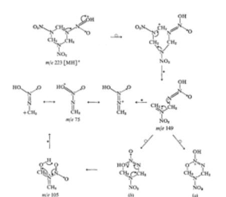

Scheme 1. Rationalization of the CI(CH₄) spectrum of 1,3,5-trinitro-1,3,5-triazacyclohexane (RDX).

1975
C. Fenselau and I. Jardine use charge exchange ionization to analyze various drugs[39]

1975
M. A. Haney and J. F. Gallagher use spark source-MS (SSMS) to differentiate bullets based on their elemental composition.[99]

1977
In *US vs Brown*, scientists from the McCrone Institute use an ion microprobe mass analyzer (AKA secondary ion mass spectrometry, SIMS) to compare the trace elements in human hair; technique found to be admissible after appeal, but the ultimate conclusions were not.[124]

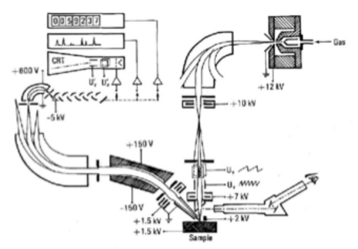

1978
To help enforce federal regulations, A. Blum et al. use GC-MS with a negative ion atmospheric pressure chemical ionization (APCI) source to identify metabolites of a flame retardant in the urine of children.[44]

1978
R. W. Kondrat and R. G. Cooks introduce mass-analyzed ion kinetic energy spectrometer (MIKES) for the tandem MS of compounds from mixtures. Demonstrate the approach to identify drugs in various matrices, including cocaine from coca leaves.[54]

1978
In *American Meat Institute vs Hon. Bergland* et al. GC-MS is used to confirm GC-thermionic emission analysis (TEA) results for nitrosamines in cooked bacon.[43]

1978
J. Locke et al. demonstrate SSMS for classification of small glass fragments.[131]

1978
Judge finds MS evidence admissible for the identification of curare (of poison arrow fame) in several exhumed bodies.[42]

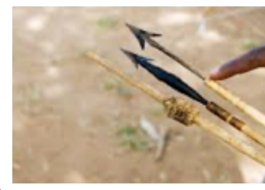

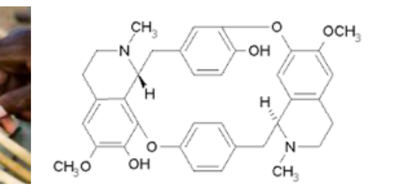

1990
A. Zurhaar and L. Mullings apply inductively-coupled plasma-mass spectrometry (ICP-MS) to forensic analysis of glass samples[135]

1994
To combat doping in sports, Becchi et al. use GC-IRMS to distinguish exogenous and endogenous testosterone in human urine.[63]

1997
D. A. Sutherland uses GC-MS/MS to detect trace gasoline residues in casework with an elevated background of weathered fuel oil.[145]

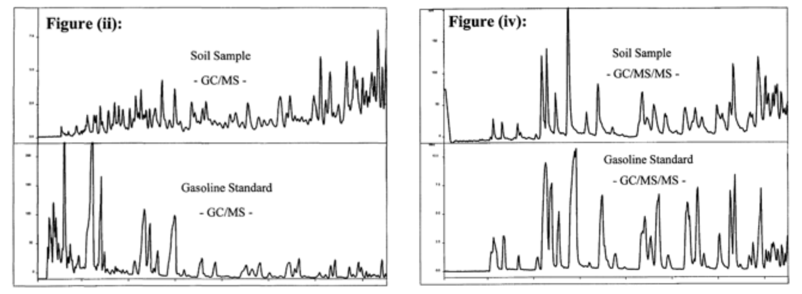

2003
R. B. Cody and J. A. Laramee file invention disclosure for direct analysis in real time (DART) for the direct analysis of non-polar molecules, including drugs and explosives, from ambient surfaces. Publish first results with H. D. Durst in 2005.[35]

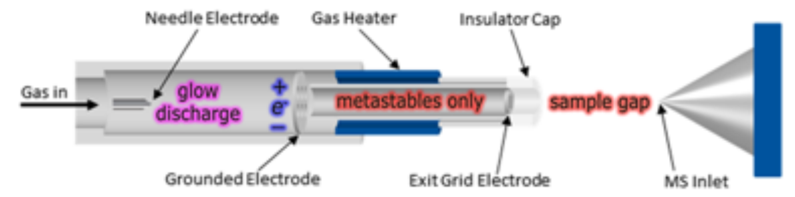

1950 1960 1970 1980 1990 2000 2010

History of Forensic Mass Spectrometry | ASMS History Committee Poster, 2020 Glen P. Jackson[1*] and Mark A. Barkett[2] [1]Department of Forensic and Investigative Science, West Virginia University [2]Dover Chemical Corporation

References for: History of Forensic Mass Spectrometry
ASMS History Committee Poster, 2020

Glen P. Jackson[1*] and Mark A. Barkett[2]
[1*]Department of Forensic and Investigative Science, West Virginia University, Morgantown, WV, USA, 26506; t 304-293-9236; e glen.jackson@mail.wvu.edu
[2]Dover Chemical Corporation

1. Yinon, J., Forensic applications of mass spectrometry. *Mass Spectrom. Rev.* **1991,** *10* (3), 179-224.
2. Liu, R. H., Gadzala, D. E., *Handbook of drug analysis: Applications in forensic and clinical laboratories*. ACS: Washington, DC, **1997**.
3. Hoffmann, W. D., Jackson, G. P., Forensic Mass Spectrometry. *Annu Rev Anal Chem (Palo Alto Calif)* **2015**.
4. *Strengthening forensic science in the United States: A path forward*. National research council: Committee on identifying the needs of the forensic sciences community; committee on applied and theoretical statistics, **2009**.
5. Careri, M., Mangia, A., Validation and qualification: the fitness for purpose of mass spectrometry-based analytical methods and analytical systems. *Anal. Bioanal. Chem.* **2006,** *386* (1), 38-45.
6. Baldwin, R., Bethem, R., Boyd, R., Budde, W., Cairns, T., Gibbons, R., Henion, J., Kaiser, M., Lewis, D., Matusik, J., Sphon, J., Stephany, R., Trubey, R., Report: 1996 ASMS Fall Workshop: Limits to Confirmation, Quantitation, and Detection *J. Am. Soc. Mass Spectrom.* **1997,** *8* (11), 1180-1190.
7. Bethem, R. A., Boyd, R. K., Mass spectrometry in trace analysis. *J. Am. Soc. Mass Spectrom.* **1998,** *9* (6), 643-648.
8. Bethem, R., Boison, J., Gale, J., Heller, D., Lehotay, S., Loo, J., Musser, S., Price, P., Stein, S., Establishing the fitness for purpose of mass spectrometric methods. *J. Am. Soc. Mass Spectrom.* **2003,** *14* (5), 528-541.
9. Matthew R. Duros, Burch, A. M., Wals, K., Tiry, E. *Publicly Funded Forensic Crime Laboratories: Resources and Services, 2014*; US DOJ, Burea of Justice Statistics: **2016**.
10. Bleakney, W., A new method of positive ray analysis and its application to the measurement of ionization potentials in mercury vapor. *Phys. Rev.* **1929,** *34*, 157-160.
11. Bleakney, W., Condon, E. U., Smith, L. G., Ionization and Dissociation of Molecules by Electron Impact. *J. Phys. Chem.* **1937,** *41* (2), 197-208.
12. Gelpi, E., From large analogical instruments to small digital black boxes: 40 years of progress in mass spectrometry and its role in proteomics. Part I 1965-1984. *J. Mass Spectrom.* **2008,** *43* (4), 419-435.
13. Gohlke, R. S., McLafferty, F. W., Early gas chromatography/mass spectrometry. *J. Am. Soc. Mass Spectrom.* **1993,** *4* (5), 367-371.
14. Beynon, J. H., The use of the mass spectrometer for the identification of organic compounds *Microchimica Acta* **1956,** *44* (1-3), 437.

15. Beynon, J. H., Qualitative Analysis of Organic Compounds by Mass Spectrometry. *Nature* **1954,** *174* (4433), 735-737.
16. Mclafferty, F. W., Mass Spectrometric Analysis - Broad Applicability to Chemical Research. *Anal. Chem.* **1956,** *28* (3), 306-316.
17. Biemann, K., Seibl, J., Application of Mass Spectrometry to Structure Problems .2. Stereochemistry of Epimeric, Cyclic Alcohols. *J. Am. Chem. Soc.* **1959,** *81* (12), 3149-3150.
18. Blossey, E. C., Budzikiewicz, H., Ohashi, M., Fodor, G., Djerassi, C., Mass spectrometry in structural and stereochemical problems. XXXIX. Tropane alkaloids. *Tetrahedron* **1964,** *20* (3), 585-95.
19. Budzikiewicz, H., Alpin, R. T., Lightner, D. A., Djerassi, C., Mechoulam, R., Gaoni, Y., Massenspektroskopie und IHRE anwendung auf strukturelle und stereochemische probleme--LXVIII: Massenspektroskopische untersuchung der inhaltstoffe von haschisch. *Tetrahedron* **1965,** *21* (7), 1881-1888.
20. Biemann, K., Friedmann, M., Application of Mass Spectrometry to Structure Problems:. V. Iboga Alkaloids. *J. Am. Chem. Soc.* **1961,** *83* (23), 4805-4810.
21. Biemann, K., Friedmannspiteller, M., Mass Spectrometric Evidence for the Structure of Iboxygaine and Its Tosylate. *Tetrahedron Letters* **1961,** (2), 68-71.
22. Djerassi, C., Biemann, K., Shoolery, J. N., Gilbert, B., Johnson, L. F., Alkaloid Studies .26. Constitution of Pyrifolidine. *Experientia* **1961,** *17* (4), 162-163.
23. Martin, R. J., Alexander, T. G., Analytical procedures used in FDA laboratories for the analysis of hallucinogenic drugs. *J. AOAC Int.* **1968,** *51*, 159-163.
24. Coutts, R. T., Locock, R. A., Identification of medicinal barbiturates by means of mass spectrometry. *J. Pharm. Sci.* **1968,** *57* (12), 2096-2100.
25. Bellman, S. W., Mass spectral identification of some hallucinogenic drugs. *J. AOAC Int.* **1968,** *51* (1), 164-178.
26. Bellman, S. W., Turcan, J. W., Kram, T. C., Spectrometric forensic chemistry of hallucinogenic drugs. *J. Forens. Sci.* **1970,** *15* (2), 261-286.
27. Nigam, I. C., Holmes, J. L., Mass spectrometry of lysergic acid diethylamide. *J. Pharm. Sci.* **1969,** *58* (4), 506-507.
28. Inoue, T., Nakahara, Y., Niwaguch.T, Studies on lysergic-acid diethylamide and related compounds. 2. Mass-spectra of lysergic-acid derivatives. *Chem. Pharm. Bull.* **1972,** *20* (2), 409-411.
29. Althaus, J., Biemann, K., Biller, J., Donaghue, P., Evans, D., Förster, H., Hertz, H., Hignite, C., Murphy, R., Preti, G., Reinhold, V., Identification of the drug Darvon and its metabolites in the urine of a comatose patient using a gas chromatograph-mass. Spectrometer-computer system. *Cell. Molec. Life Sciences* **1970,** *26* (7), 714-717.
30. Law, N. C., Aandahl, V., Fales, H. M., Milne, G. W. A., Identification of dangerous drugs by mass spectrometry. *Clin. Chim. Acta* **1971,** *32* (2), 221-228.
31. Skinner, R. F., Gallaher, E. J., Knight, J. B., Bonelli, E. J., The gas chromatograph-mass spectrometer as a new and important tool in forensic toxicology. *J. Forens. Sci.* **1972,** *17* (2), 189-198.
32. Green, D. E., Automated detection of abused drugs by direct mass fragmentography. *Proceedings of the Western Pharmacology Society* **1972,** *15*, 74-77.
33. Cooks, R. G., Ouyang, Z., Takats, Z., Wiseman, J. M., Ambient mass spectrometry. *Science* **2006,** *311* (5767), 1566-1570.

34. Takats, Z., Wiseman, J. M., Gologan, B., Cooks, R. G., Mass spectrometry sampling under ambient conditions with desorption electrospray ionization. *Science* **2004,** *306* (5695), 471.
35. Cody, R. B., Laramee, J. A., Durst, H. D., Versatile new ion source for the analysis of materials in open air under ambient conditions. *Anal. Chem.* **2005,** *77* (8), 2297.
36. *The New York Times* July 12, **1973**.
37. Munson, M. S. B., Field, F. H., Chemical ionization mass spectrometry. I. General introduction. *J. Am. Chem. Soc.* **1966,** *88* (12), 2621-2630.
38. Saferstein, R., Chao, J.-M., Identification of drugs by chemical ionization mass spectroscopy. *J. AOAC Int.* **1973,** *56* (5), 1234-8.
39. Jardine, I., Fenselau, C., Charge exchange mass spectra of morphine and tropane alkaloids. *Anal. Chem.* **1975,** *47* (4), 730-3.
40. Fenselau, C., Gas Chromatography Mass Spectrometry: A Report on the State of the Art. *Appl. Spec.* **1974,** *28*, 305-318.
41. Citizens against toxic sprays, Inc., et al., Plaintiffs, v. Bob Bergland, Secretary, United States Department of Agriculture, et al., Defendants, Industrial Forestry Association, Defendant-Intervenor. United states district court for the district of Oregon: **1977**.
42. Bird, D., Detection of curare in Jascalevich murder trail. *New York Times* June 23, **1978**, p 3.
43. American Meat Institute, Plaintiff, v. The Honorable Robert S. Bergland et al., Defendants. United States District Court for the District of Columbia: **1978**.
44. Blum, A., Gold, M., Ames, B., Jones, F., Hett, E., Dougherty, R., Horning, E., Dzidic, I., Carroll, D., Stillwell, R., Thenot, J., Children absorb tris-BP flame retardant from sleepwear: urine contains the mutagenic metabolite, 2,3-dibromopropanol. *Science* **1978,** *201* (4360), 1020-1023.
45. Cohn, V., Studies say Tris in old pajamas absorbed in children's skin. *The Washington Post* **1978**.
46. Bigger, P. J., Urinalysis - issues and applications. *Federal Probation* **1979,** *43* (4), 23-37.
47. Stein, B., Laessig, R. H., Indriksons, A., An Evaluation of Drug Testing Procedures Used by Forensic Laboratories and the Qualification of their Analysts. *Wisc. L. Rev.* **1973**, 727-789.
48. Guthrie, R. D., McCarthy, J. F., Mass spectra of φ-pelletierine, 9-methyl-3-oxagranatan-7-one, and 9-methyl-3-oxagranatan-7α-ol. *J. Chem. Soc. (C)* **1966,** *13*, 1207-9.
49. Dewhurst, J. E., Kaminski, J. J., Supple, J. H., Mass spectra of some tropane and tropidine derivatives. *J. Heterocycl. Chem.* **1972,** *9* (3), 507-11.
50. Moore, J. M., Identification of cis- and trans-cinnamoylcocaine in illicit cocaine seizures. *J. AOAC Int.* **1973,** *56* (5), 1199-205.
51. Fales, H. M., Milne, G. W. A., Law, N. C., Mass spectra of two tropanes: cocaine and scopolamine, 2β-methoxycarbonyl-3β-benzoyloxytropane. *Arch. Mass Spectral Data* **1971,** *2* (4), 654-7.
52. Suzuki, R., Murata, M., Kamei, K., Momose, A., Studies on doping test by gas chromatography-mass spectrometry. IV. Gas chromatography and mass spectrometry of local anesthetics. *Yakugaku Zasshi* **1973,** *93* (7), 942-7.
53. Kirchgessner, W. G., DiPasqua, A. C., Anderson, W. A., Delaney, G. V., Drug identification by the application of gas chromatography/time-of-flight mass spectrometer technique. *J. Forensic Sci.* **1974,** *19*, 313-16.

54. Kondrat, R. W., Cooks, R. G., Direct analysis of mixtures by mass spectrometry. *Anal. Chem.* **1978,** *50* (1), 81A-92A.
55. State of Wisconsin, Plaintiff-Respondent, v. Daniel Barnes, Defendant-Appellant. Court of Appeals of Wisconsin, District II: **1979**.
56. State ex rel. Huser, Petitioner-Appellant, v. Rasmussen, Sheriff of Green Lake County, Respondent. Supreme Court of Wisconsin: **1978**.
57. State of Wisconsin, Plaintiff-Respondent, v. Cleoothur McNeal, Defendant-Appellant. Court of Appeals of Wisconsin: **1980**.
58. United States of America, Plaintiff-Appellee, v. Patricia Ann Larry, aka Kim, Defendant-Appellant. United States court of Appeals for the Tenth Circuit: **1975**.
59. United States of America, Plaintiff-Appellee, v. Martin ross, Defendant-Appellant. United states court of appeals for the second circuit: **1983**.
60. United States of America, Plaintiff-Appellee, v. Gary Dale Posey, Defendant-Appellant. United states court of appeals, tenth circuit: **1981**.
61. Hemmersbach, P., History of mass spectrometry at the Olympic Games. *J. Mass Spectrom.* **2008,** *43* (7), 839-853.
62. Todd, J., Todd, T., Significant events in the history of drug testing and the Olympic movement: 1960-1999. In *Doping in elite sport: the politics of drugs in the Olympic movement* Wilson, W., Ed. Human Kinetics Publishers: Champaign, **2001**; p 65.
63. Becchi, M., Aguilera, R., Farizon, Y., Flament, M. M., Casabianca, H., James, P., Gas-Chromatography Combustion Isotope Ratio Mass-Spectrometry Analysis of Urinary Steroids to Detect Misuse of Testosterone in Sport. *Rapid. Commun. Mass Spectrom.* **1994,** *8* (4), 304-308.
64. Nicol, J. D., Police Science Technical Abstracts and Notes. *J. Crim. Law Criminol.* **1959,** *40*, 109-112.
65. Virgle L. Miller, Appellant v. The State of Texas, Appellee. Court of Criminal Appeals of Texas: **1978**.
66. State of Montana, Plaintiff and Respondent, v. James Burtchett, Defendant and Appellant. Supreme Court of Montana: **1974**.
67. United States, Appellee v Larry J. Harvey, Private, Clarance R. Lee, Specialist Four, and Arnold E. Taylor, Private, U.S. Army, Appellants. United States Court of Military Appeals: **1971**.
68. Zoro, J. A., Hadley, K., Organic Mass Spectrometry in Forensic Science. *J. Forens. Sci. Soc.* **1976,** *16* (2), 103-114.
69. Quinn, C. C., Cartridge discharge residue contamination - the search for the source. *Science & Justice* **1998,** *38* (2), 81-84.
70. Hall, D., Fairley, M., A single approach to the recovery of DNA and firearm discharge residue evidence. *Science & Justice* **2004,** *44* (1), 15-19.
71. Turkel, H., Lipman, J., Unreliabillty of Dermal Nitrate Test for Gunpowder. *J. Crime Law Criminol. Polit. Sci.* **1955,** *281* (282).
72. Vincent, J. M., *Gunshot Wounds: Practical Aspects of Firearms, Ballistics, and Forensic Techniques*. Elsevier: New York, **1999**.
73. Commonwealth v. Westwood, Appellant. Supreme Court of Pennsylvania: **1936**.
74. Giannelli, P. C., 137 Military Law Review 167. *Scientific Evidence in Criminal Prosecutions* **1992**.
75. Cowan, M., Purdon, P., A Study of the "Paraffin Test". *J. Forens. Sci.* **1967,** *12* (1), 19-35.

76. Schwoeble, A. J., Exline, D. L., *Current Methods in Forensic Gunshot Residue Analysis*. CRC Press: Washington D.C., **2000**.
77. Sharma, S. P., Lahiri, S. C., A preliminary investigation into the use of FTIR microscopy as a probe for the identification of bullet entrance holes and the distance of firing. *Science & Justice* **2009,** *49* (3), 197-204.
78. Bakowska, E., Harrsch, P., Gluodenis, T., Jr., Analysis of Gunshot Residue by ICP-MS. Technologies, A., Ed. Agilent Technologies: Santa Clara, **2001**.
79. Perret, D., Marchese, S., Gentili, A., Curini, R., Terracciano, A., Bafile, E., Romolo, F., LC–MS–MS Determination of Stabilizers and Explosives Residues in Hand-Swabs. *Chromatographia* **2008,** *68* (7), 517-524.
80. Cotte-Rodriguez, I., Chen, H., Cooks, R. G., Rapid trace detection of triacetone triperoxide (TATP) by complexation reactions during desorption electrospray ionization. *Chem. Comm.* **2006,** (9), 953-955.
81. Takats, Z., Cotte-Rodriguez, I., Talaty, N., Chen, H. W., Cooks, R. G., Direct, trace level detection of explosives on ambient surfaces by desorption electrospray ionization mass spectrometry. *Chem. Comm.* **2005,** (15), 1950-1952.
82. Michael David Dowland, Plaintiff and Appellant, v. Lyman Products for Shooters, a corporation, Euroarms, The Leisure Group, Inc., and ABC corporations 1 through 10, Defendants and Respondents. Supreme Court of Utah: **1982**.
83. ASTM, E1588-17 Standard Practice for Gunshot Residue Analysis by Scanning Electron Microscopy/Energy Dispersive X-Ray Spectrometry. ASTM: **2017**.
84. Yinon, J., *The analysis of explosives* 1st ed ed.; Pergamon Press: Oxford ; New York :, **1981**; p xii, 310 p. ;.
85. Yinon, J., Identification of Explosives by Chemical Ionization Mass-Spectrometry Using Water as Reagent. *Biomed Mass Spectrom* **1974,** *1* (6), 393-396.
86. Gillis, R. G., Lacey, M. J., Shannon, J. S., Chemical Ionization Mass-Spectra of Explosives. *Org Mass Spectrom* **1974,** *9* (3), 359-364.
87. Pate, C. T., Mach, M. H., Analysis of Explosives Using Chemical Ionization Mass-Spectroscopy. *Int J Mass Spectrom* **1978,** *26* (3), 267-277.
88. , Committee on Assessment of Security Technologies for Transportation *Opportunities to Improve Airport Passenger Screening with Mass Spectrometry*; National Research Council: Washington, **2004**.
89. Chesson, L. A., Howa, J. D., Lott, M. J., Ehleringer, J. R., Development of a methodological framework for applying isotope ratio mass spectrometry to explosive components. *Forensic Chemistry* **2016,** *2*, 9-14.
90. Howa, J. D., Barnette, J. E., Chesson, L. A., Lott, M. J., Ehleringer, J. R., TATP isotope ratios as influenced by worldwide acetone variation. *Talanta* **2018,** *181*, 125-131.
91. Howa, J. D., Lott, M. J., Chesson, L. A., Ehleringer, J. R., Carbon and nitrogen isotope ratios of factory-produced RDX and HMX. *Forensic Sci Int* **2014,** *240*, 80-7.
92. Howa, J. D., Lott, M. J., Chesson, L. A., Ehleringer, J. R., Isolation of components of plastic explosives for isotope ratio mass spectrometry. *Forensic Chemistry* **2016,** *1*, 6-12.
93. Howa, J. D., Lott, M. J., Ehleringer, J. R., Observations and sources of carbon and nitrogen isotope ratio variation of pentaerythritol tetranitrate (PETN). *Forensic Sci Int* **2014,** *244*, 152-7.

94. Howa, J. D., Lott, M. J., Ehleringer, J. R., Isolation and stable nitrogen isotope analysis of ammonium ions in ammonium nitrate prills using sodium tetraphenylborate. *Rapid Commun Mass Spectrom* **2014,** *28* (13), 1530-4.
95. Benson, S. J., Lennard, C. J., Hill, D. M., Maynard, P., Roux, C., Forensic Analysis of Explosives Using Isotope Ratio Mass Spectrometry (IRMS); Part 1: Instrument Validation of the DELTA^{XP} IRMS for Bulk Nitrogen Isotope Ratio Measurements. *J. Forens. Sci.* **2010,** *55* (1), 193-204.
96. Ehleringer, J. R., Matheson Jr., S. M., Stable Isotopes and Courts. *Utah Law Review No. 2* **2010**, 385-442.
97. Benson, S., Lennard, C., Maynard, P., Roux, C., Forensic applications of isotope ratio mass spectrometry: A review. *Forens. Sci. Int.* **2006,** *157* (1), 1-22.
98. Muccio, Z., Jackson, G. P., Isotope Ratio Mass Spectrometry. *Analyst* **2009,** *134* (2), 213-22.
99. Haney, M. A., Gallagher, J. F., Differentiation of bullets by spark source mass spectrometry. *J. Forens. Sci.* **1975,** *20* (3), 484.
100. Houk, R. S., Fassel, V. A., Flesch, G. D., Svec, H. J., Gray, A. L., Taylor, C. E., Inductively Coupled Argon Plasma as an Ion-Source for Mass-Spectrometric Determination of Trace-Elements. *Anal. Chem.* **1980,** *52* (14), 2283.
101. Shane Layton Ragland, Appellant v. Commonwealth of Kentucky, Appellee. Supreme Court of Kentucky: **2006**.
102. Ammann, A. A., Inductively coupled plasma mass spectrometry (ICP MS): a versatile tool. *J. Mass Spectrom.* **2007,** *42* (4), 419-427.
103. Flesch, P., Rothman, S., Isolation of an iron pigment from human red hair. *J. Invest. Dermatol.* **1945,** *6* (4), 257-270.
104. Nickerson, M., Relation between black and red melanin pigment in feathers. . *Physiological Zoology* **1946,** *19* (1), 66-77.
105. Rothman, S., Schaaf, F., Die Chemie der Haut. In *Jadasshon, Handb. d. Haut, u. Geschlechtskr*, Springer: Berlin, **1929**; pp 161-377.
106. Jackson, S. H., Determination of iron in biological material. *Industrial and Engineering Chemistry, Analytical Edition* **1938,** *10* (6), 302-304.
107. Parker, W. E., Griffin, F. P., Some Observations on the Determination of Iron and Copper in Biological Material by Photoelectric Colorimetry. *Can. J. Res.* **1939,** *B17* (2), 66-70.
108. Gerber, L., Claassen, R., Boruff, C., Photometric Determination of Copper and Iron in Distilled Liquors. *Ind. Eng. Chem. Anal. Ed.* **1942,** *14* (4), 364-366.
109. Koenig, R. A., Johnson, C. R., Spectrophotometric detemrination of ironII; Use of α-α' Bipyridine. . *J. Biol. Chem.* **1941,** *143*, 159-163.
110. Hill, R., A Method for the Estimation of Iron in Biological Material. *Proc. Royal Soc. London B.* **1930,** *107* (750), 205-214.
111. Flesch, P., The role of copper in mammalian pigmentation. *Proc. Soc. Experiment. Biol. Med.* **1949,** *70*, 79.
112. Sarata, U., Copper in pigmentation of skin and hair. *Jpn. J. Med. Sci.* **1935,** *3*, 79.
113. Yosikawa, H., Copper in black and white hairs of aged people. *Jpn. J. Med. Sci.* **1937,** *3*, 195-198.
114. Saccardi, P., Guiliani, G., The copper content in hair and feathers of animals of different colors. *Biochimica e Terapia Sperimentale* **1935,** *22*, 169-172.

115. Rice, E. W., Goldstein, N. P., Copper content of hair and nails in Wilson's disease (hepatolenticular degeneration). *Metabolism* **1961,** *10*, 1085-1087.
116. Martin, G. M., Copper content of hair and nails of normal individuals and of patients with hepato-lenticular degeneration. *Nature* **1964,** *202*, 903-904.
117. Dutcher, T. F., Rothman, S., *J. Invest. Dermatol.* **1951,** *17*, 65.
118. Kopita, L., Byers, R. K., Schwachman, H., Lead in hair of children with chronic lead poisoning. *New England J. Chem.* **1967,** *276*, 949.
119. Harrison, W. W., Yurachek, J. P., Benson, C. A., The determination of trace elements in human hair by atomic absorption spectroscopy. *Clin. Chim. Acta* **1969,** *23* (1), 83-91.
120. Yurachek, J. P., Clemena, G. G., Harrison, W. W., Analysis of human hair by spark source mass spectrometry. *Anal. Chem.* **1969,** *41* (12), 1666-1668.
121. Bate, L. C., Dyer, F., Trace elements in human hair. *Nucleonics* **1965,** *23* (10), 74-81.
122. Castaing, R., Slodzian, G., Microanalyse parmission ionique secondaire (Microanalysis by secondary ion emission). *Journal De Microscopie* **1962,** *1*, 395.
123. Andersen, C. A., Hinthorne, J. R., Ion Microprobe Mass Analyzer. *Science* **1972,** *175* (4024), 853-860.
124. United States of America, Plaintiff-Appellee, v. Hayward Leslie Brown, Defendant-Appellant. United States court of Appeals for the Sixth Circuit: **1977**.
125. Saferstein, R., Manura, J. J., Pyrolysis Mass Spectrometry - A New Forensic Science Technique. *J. Forens. Sci.* **1977,** *22* (4), 748-756.
126. Hughes, J. C., Wheals, B. B., Whitehouse, M. J., Pyrolysis Mass-Spectrometry - Technique of Forensic Potential. *Forensic Sci* **1977,** *10* (3), 217-228.
127. Scientific Working Group on Materials Analysis (SWGMAT), Forensic paint analysis and comparison guidelines. **1999**.
128. Hobbs, A. J., Almirall, J. R., Trace elemental analysis of automotive paints by laser ablation-inductively coupled plasma-mass spectrometry. *Anal. Bioanal. Chem.* **2003,** *376*, 1265-1271.
129. Farmer, N., Meier-Augenstein, W., Lucy, D., Stable isotope analysis of white paints and likelihood ratios. *Science & Justice* **2009,** *49* (2), 114-119.
130. Maynard, P., Gales, K., Roux, C., Lennard, C. J., Adhesive Tape Analysis: Establishing the Evidential Value of Specific Techniques. *J. Forens. Sci.* **2001,** *46* (2), 280-287&.
131. Locke, J., Boase, D., Smalldon, K. W., The Use of Spark Source Mass Spectrometry for the Analysis and Classification of Small Glass Fragments. *J. Forens. Sci. Soc.* **1978,** *18* (1-2), 123-131.
132. Houk, R. S., Inductively Coupled Argon Plasma as an Ion Source for Mass Spectrometric Determination of Trace Elements. *Anal. Chem.* **1980,** *52* (14), 2283-2289.
133. Duckworth, D. C., Bayne, C. K., Morton, S. J., Almirall, J., Analysis of variance in forensic glass analysis by ICP-MS: Variance within the method. *J. Anal. Atom. Spectrom.* **2000,** *15* (7), 821-828.
134. Parouchais, T., Warner, I. M., Palmer, L. T., Kobus, H., The analysis of small glass fragments using inductively coupled plasma mass spectrometry. *J. Forens. Sci.* **1996,** *41* (3), 351-360.
135. Zurhaar, A., Mullings, L., Characterisation of forensic glass samples using inductively coupled plasma mass spectrometry. *J. Anal. Atom. Spectrom.* **1990,** *5* (7), 611-617.

136. ASTM, E2927-16E1: Standard Test Method for Determination of Trace Elements in Soda-Lime Glass Samples Using Laser Ablation Inductively Coupled Plasma Mass Spectrometry for Forensic Comparisons. **2017**.
137. Trejos, T., Montero, S., Almirall, J. R., Analysis and comparison of glass fragments by laser ablation inductively coupled plasma mass spectrometry (LA-ICP-MS) and ICP-MS. *Anal. Bioanal. Chem.* **2003,** *376* (8), 1255-1264.
138. English, J. M., Forensic Science in Criminal Prosecution. *Anal. Chem.* **1970,** *42* (13), 40A-48A.
139. Brown, R., The Mass Spectrometry of Hydrocarbons. In *Hydrocarbon Analysis*, Smith, H., Ed. ASTM Int.: West Conshohocken, PA, **1965**; pp 68-102.
140. Fales, H. M., Milne, G. W. A., Axenrod, T., Identification of barbiturates by chemical ionization mass spectrometry. *Anal. Chem.* **1970,** *42* (12), 1432-1435.
141. Comprehensive Drug Abuse Prevention and Control Act. In *91-513.*, L., U. P., Ed. **1970**.
142. Bommer, P., Moser, H., Stichler, W., Trimborn, P., Vetter, W., Determination of Origin of Drugs by Measuring Natural Isotope Contents - D-H and C-13-C-12 Ratios of Some Diazepam Samples. *Z Naturforsch C* **1976,** *31* (3-4), 111-114.
143. Ohio State Racing Commisssion, Plaintiff-Appellee, v., James A. GarrisonDefendant-Appellant. Court of Appeals of Ohio, Tenth Appellate District, Franklin County: **1980**.
144. Daubert v. Merrell Dow Pharmaceuticals. United States Supreme Court: **1993**; Vol. 113 S. Ct. .
145. Sutherland, D. A., The Analysis of Fire Debris Samples by GC/MS/MS. *Canadian Society of Forensic Science Journal* **1997,** *30* (4), 185-189.
146. Ehleringer, J. R., Cooper, D. A., Lott, M. J., Cook, C. S., Geo-location of heroin and cocaine by stable isotope ratios. *Forens. Sci. Int.* **1999,** *106* (1), 27-35.
147. Meier-Augenstein, W., Fraser, I., Forensic isotope analysis leads to identification of a mutilated murder victim. *Science & Justice* **2008,** *48* (3), 153-159.
